# Genome-Wide Association Analysis of the Anthocyanin and Carotenoid Contents of Rose Petals

**DOI:** 10.3389/fpls.2016.01798

**Published:** 2016-12-06

**Authors:** Dietmar F. Schulz, Rena T. Schott, Roeland E. Voorrips, Marinus J. M. Smulders, Marcus Linde, Thomas Debener

**Affiliations:** ^1^Abteilung Molekulare Pflanzenzüchtung, Institute for Plant Genetics, Leibnitz University HannoverHannover, Germany; ^2^Wageningen University and Research Plant Breeding, Wageningen University and Research CentreWageningen, Netherlands

**Keywords:** petal color, anthocyanin, carotenoid, genome wide association study, tetraploid roses

## Abstract

Petal color is one of the key characteristics determining the attractiveness and therefore the commercial value of an ornamental crop. Here, we present the first genome-wide association study for the important ornamental crop rose, focusing on the anthocyanin and carotenoid contents in petals of 96 diverse tetraploid garden rose genotypes. Cultivated roses display a vast phenotypic and genetic diversity and are therefore ideal targets for association genetics. For marker analysis, we used a recently designed Axiom SNP chip comprising 68,000 SNPs with additionally 281 SSRs, 400 AFLPs and 246 markers from candidate genes. An analysis of the structure of the rose population revealed three subpopulations with most of the genetic variation between individual genotypes rather than between clusters and with a high average proportion of heterozygous loci. The mapping of markers significantly associated with anthocyanin and carotenoid content to the related *Fragaria* and *Prunus* genomes revealed clusters of associated markers indicating five genomic regions associated with the total anthocyanin content and two large clusters associated with the carotenoid content. Among the marker clusters associated with the phenotypes, we found several candidate genes with known functions in either the anthocyanin or the carotenoid biosynthesis pathways. Among others, we identified a glutathione-S-transferase, 4CL, an auxin response factor and F3'H as candidate genes affecting anthocyanin concentration, and CCD4 and Zeaxanthine epoxidase as candidates affecting the concentration of carotenoids. These markers are starting points for future validation experiments in independent populations as well as for functional genomic studies to identify the causal factors for the observed color phenotypes. Furthermore, validated markers may be interesting tools for marker-assisted selection in commercial breeding programmes in that they provide the tools to identify superior parental combinations that combine several associated markers in higher dosages.

## Introduction

Rose is one of the most economically important ornamental crops and is sold as cut flowers, pot roses and garden roses. The genus *Rosa* comprises a vast amount of genetic resources represented by more than 100 wild species as well as more than 30,000 mostly tetraploid varieties bred for ornamental purposes (Gudin, 2000; Wissemann, 2003). Cultivated tetraploid rose genomes are complex mixtures of at least 10 species that have been used in ornamental rose breeding for more than two centuries (Gudin, 2000; Zhang et al., 2013). As a result, rose is highly diverse in many morphological and physiological characteristics. Despite its commercial importance as an ornamental plant, genomic resources for rose research and breeding remain scarce, and to date, no genome sequence is available. At the diploid level, genetic maps have been constructed, and a number of monogenic and quantitative traits have been localized on these maps (Debener and Linde, 2009; Spiller et al., 2011). However, as most diploid populations have derived from a few diploid genotypes, genetic variability is low for most horticultural traits. Therefore, these traits can only be analyzed at the tetraploid level. In tetraploid varieties, several monogenic traits have been analyzed, but only few QTL have been described, mostly by analyses of biparental populations (Debener and Linde, 2009; Spiller et al., 2011).

The esthetic features of the rose flower are of central importance for the ornamental quality of rose cultivars; therefore, commercial breeding pays special attention to floral characteristics. Flower traits, e.g., the number and color of petals, were among the first traits investigated in genetic studies (De Vries and Dubois, 1978; Debener, 1999, 2003).

The anthocyanin concentration in cells of rose petals is a major determinant of red and pink color variants, although the final hues are influenced by several other factors as e.g., pH, copigments, metal ions, types of glycosylation, etc. (Jay et al., 2003; Grotewold, 2006; Tanaka et al., 2008). Although single loci influencing color variation have been identified, other researchers have described a quantitative inheritance of the anthocyanin content (Cardoso et al., 2012; Cericola et al., 2014; Henz et al., 2015).

The levels of carotenoids, which produce yellow colors, are influenced by biotic and abiotic factors, including the developmental stage, the environment and stress (Eugster and Märki-Fischer, 1991; Deli et al., 1998; Kishimoto et al., 2004). In fully opened flowers of Ipomoea, the chromoplast-type carotenoids are ß-cryptoxanthin, zeaxanthin, and ß-carotene, whereas lutein, violaxanthin and ß-carotene are predominant in the early stage of petal development, and the same compounds were found in the leaves (Yamamizo et al., 2010).

### QTL mapping and GWAS

All of the QTLs studied to date in rose have been mapped in biparental populations (Crespel et al., 2002; Linde et al., 2006; Spiller et al., 2011; Moghaddam et al., 2012; Roman et al., 2015) using AFLP and microsatellite markers. Tetraploid populations derived from crosses between ornamental varieties display complex patterns of inheritance that complicate not only genetic analysis but also map construction and require many more markers (Bourke et al., 2015).

Association studies offer two main advantages over QTL studies based on biparental populations: a larger number of alleles per locus and a higher resolution of trait-marker associations due to a higher rate of recombination. In association genetics, genotyping can be restricted to candidate genes likely involved in the expression of the traits under study or markers covering the whole genome in genome-wide association studies (GWAS). Few studies cover polyploids, and very few GWAS have been performed on highly heterozygous polyploids, such as potato (D'hoop et al., 2010; Lindqvist-Kreuze et al., 2014), switchgrass (Lu et al., 2013) and cotton (Abdurakhmonov et al., 2009). Rose is an interesting ornamental crop for association studies because its cultivars are extremely polymorphic, and many traits can be studied simultaneously in populations of moderate size.

Recently, an analysis of a large collection of rose ESTs and the development of an Axiom SNP array was described (Koning-Boucoiran et al., 2015). These resources are a significant extension of the genomic resources available for roses because they now permit the highly reproducible genotyping of rose genomes with approximately 68,000 SNP markers each represented by two probes. Hence, sufficient numbers of markers are now available for GWAS in tetraploid rose.

### Aims of the present study

The aim of the present study was to exploit the enormous biodiversity of cultivated roses in flower-related traits for an analysis of the underlying genetic factors, focusing on the contents of anthocyanins and carotenoids, which are the main components of the rose petal color. This was accomplished using a combination of association genetics methods and markers on the SNP array and additional markers derived from candidate genes, SSRs and AFLPs. In addition, we tried to gain information about the variability of the genetic diversity and heterozygosity within our association panel.

## Materials and methods

### Plant material

An association panel of 96 rose cultivars with code numbers from 1 to 141 (87 tetraploid, 8 triploid, and 1 diploid) was used for the present study. Most of the cultivars were commercially available or provided by German rose-breeding companies (Table S1). Based on known pedigrees, we attempted to minimize genotypic relatedness, which can result in spurious associations, while capturing the vast diversity of phenotypic traits, including different flower colors, plant architectures, etc. Clones of each cultivar, grafted on *R. corymbifera* “Laxa,” were planted in three randomized blocks in a field at Hannover-Herrenhausen (Germany) in the spring of 2012. A second collection of cultivars was maintained under semi-controlled conditions as potted plants in three randomized blocks in a greenhouse (Federal Plant Variety Office, Hannover). The plants were initially cultivated in 3-l pots and then transferred to 7-l pots with the fertilized substrate Einheitserde T (Einheitserdewerke Gebr. Patzer, Sinntal-Altengronau, D) under natural light with a day and night temperature of 22° ± 5°C.

### Anthocyanin content of petals

Flowers were always sampled from 8 to 12 a.m. Opened buds at flower development stage 3 (Picone et al., 2004) were selected from each genotype and kept on ice until sample preparation on the same day. The anthocyanin content of petals was estimated according to Henz et al. (2015) with minor modifications. Three replicates (each 50 mg in fresh weight) from petals of each clone (3 biological replicates) were placed in 2 ml test tubes and extracted in 1 ml of methanol/HCl (99:1 v/v) (Figure S7). Following an overnight incubation (16 h) in the dark at 18°C, the total anthocyanin content in the solvent was determined based on the absorbance at 525 nm using a UV-Vis-Photometer UV mcSAFAS (Deelux Labortechnik GmbH, Germany). If necessary (E_525nm_ > 1.0), the anthocyanin extracts were diluted with the extraction solvent. Each clone was measured three times, and the overall mean was calculated for each cultivar. The anthocyanin content was recorded and evaluated in two environments: (i) in the field at Herrenhausen and (ii) in the greenhouse at the Federal Plant Variety Office, Hannover. The absorbance values were not used to calculate the levels of compounds, as this measurement involves variable mixtures of anthocyanidins.

### Carotenoid content of petals

The content of carotenoids was evaluated from all cultivars cultivated in the greenhouse at the Federal Plant Variety Office Hannover and from 20 of the cultivars in the field at Herrenhausen. The accumulation of total carotenoids in rose petals was estimated according to de Vries et al. (1974) with modification. Petals (50 mg each) were extracted with 1 ml of a mixture of petroleum ether:acetone (3:2 v/v) for 4 h at 18°C in the dark, and the carotenoids in the samples were measured spectrophotometrically at a wavelength of 442 nm. The extracts showed three characteristic absorption maxima for carotenoids at 419, 442, and 471 nm (Figure S1). These maxima suggest Violaxanthin (420, 443, and 471 nm) or Neoxanthin (420 442, and 473 nm) as components of the extract in addition to other possible compounds, respectively (Wellburn, 1994; Tinoi et al., 2006). The overlap in absorption at 442 nm is the reason that we did not determine the levels of a particular compound.

### DNA extraction

DNA was extracted from young rose leaves as described by Klie et al. (2013). The quality of DNA was checked on agarose gels, and quantification was performed using a Nanodrop 2000c spectrophotometer (PeQLab Biotechnologie GmbH, Erlangen, Germany).

### Microsatellites (SSRs)

PCR was performed in 15 μl of Williams buffer (Williams et al., 1990) containing 0.2 mM dNTP, 0.5 μM forward and reverse primers, and 1 U of DCS Taq DNA polymerase (Enzymatics, Beverly, USA). PCR conditions were as follows: initial denaturation for 5 min. at 94°C, 28 cycles of 45 s at 94°C, 45 s at 50, 55 or 60°C (Table S2), 60 s at 72°C. SSR marker bands were visually inspected and dominantly scored, and the data were transferred to a 0/1 matrix.

### AFLP

AFLP markers were generated according to the protocol of Klie et al. (2013) with 250 ng of genomic DNA. We tested 21 *Hind*III and *Mse*I primer pair combinations in the end reaction. Bands were scored dominantly and recorded in a 0/1 matrix for absence/presence of marker fragments.

### SNPs

SNPs were analyzed using the Axiom WagRhSNP array, which contains 89,893 SNPs derived from cut roses and from garden roses (Koning-Boucoiran et al., 2015). The hybridisation intensities were interpreted as tetraploid SNP dosage scores (AAAA, AAAB, AABB, ABBB, and BBBB) using fitTetra (Voorrips et al., 2011) and were used to calculate the statistics required for the association study. SNP markers that were polymorphic and scorable were used for GWAS after filtering for minor allele frequency (MAF > 0.1) and missing data (<10%). Heterozygosity was calculated as the percentage of heterozygous loci (ABBB, AABB, and ABBB) compared to the total number of loci.

### Population structure

The population structure was modeled in STRUCTURE 2.3.4 (Pritchard et al., 2000; Falush et al., 2007) with a burn-in of 10,000 cycles and varied in the number of following Markov Chain Monte Carlo (MCMC) iterations (50,000 and 100,000) and the number of AFLP (400) and microsatellite and candidate gene markers (175 and 527). The SNP markers were not used for determining the population structure. We used the implemented admixture model with correlated allele frequencies and an initial alpha of 1 and accomplished three independent runs based on 10 repeats of the simulations for each K, from *K* = 1 to 10. Then, the most likely number of subpopulations was estimated based on the method of Evanno et al. (2005) using the InP (D) value (estimated likelihood) with the software StructureHarvester (http://taylor0.biology.ucla.edu/structureHarvester/) (Earl and von Holdt, 2012).

Furthermore, a PCoA (Principal Coordinate Analysis) was performed using DARwin 5.0.158 (Perrier and Jacquemoud-Collet, 2006) with the same subset of 927 AFLP, SSR and candidate gene markers.

### Genetic diversity analysis

The genetic distance among the collection of rose genotypes was calculated with DARwin using a subset of 16,040 SNPs at the tetraploid dosage state from the filtered SNP set (MAF > 0.1 and missing data <10%). An unweighted neighbor-joining (Saitou and Nei, 1987) dendrogram was constructed based on a distance dissimilarity matrix using a bootstrap analysis with 100 repetitions.

### Kinship matrix calculation

A kinship matrix was used to establish and describe the relationship between the genotypes. Pairwise kinship coefficients were estimated using the programme SPAGeDi (Hardy and Vekemans, 2002) based on the method of Hardy (2003) using 10,000 random SNP markers at the tetraploid dosage state from the 16,040 SNPs above. The diagonal of the matrix from SPAGeDi was set to two, and negative values were set to zero (Yu et al., 2006).

### Trait-marker associations

Trait-marker association analysis was performed using the mixed linear model (MLM, K + Q) in TASSEL 3.0 (Bradbury et al., 2007). SNP markers with a minor allele frequency of less than 0.1 and with more than 10% missing data were excluded from further analysis. In TASSEL 3.0, marker allele configurations can only be used in a diploid configuration (e.g., AA, AB, or BB). Therefore, bi-allelic SNPs of the tetraploid rose cultivars were coded as diploids. For this, all possible heterozygous genotypes (AAAB, AABB, and ABBB) were coded as AB, similar to how Li et al. (2014) analyzed diploid and tetraploid Alfalfa genotypes and how Lindqvist-Kreuze et al. (2014) analyzed potato genotypes. Associations were estimated including the Q-matrix for population effects based on the output from STRUCTURE 2.3.4 (based on AFLP, microsatellite and candidate gene data) and the kinship matrix (K) calculated with SPAGeDi (based on SNP data). Bonferroni adjustments of the *p*-values were made to correct for the number of independent tests and to establish a threshold (Johnson et al., 2010). For this, a total of 19,074 independent tests (number of contigs) were assumed because a precise estimation of the real number of independent tests could not be made due to unknown linkages between most of the markers. An SNP marker was considered associated if its −log10 *p*-value was greater than 5.58.

### Statistical analysis

The nonparametric Kruskal–Wallis rank-sum test was used to identify significant differences in the mean SNP effect in groups of cultivars. Spearman rank correlation was used to test the association between the anthocyanin content in petals from greenhouse and field-grown roses. Significant differences in the means of heterozygosity in different growth types of roses were calculated using the Wilcoxon signed rank test. The data were tested for normal distribution using the Shapiro-Wilk test (α = 0.05). The data that were not normally distributed were transformed using log- and Box-Cox transformation (Wessa, 2016). The statistical calculations were performed in Excel 2007, MYSTAT 12 (Systat Software, Inc.) and QtiPlot 0.9.9 (Vasilief, 2015).

## Results

### Population structure

The population structure was analyzed based on three independent runs of STRUCTURE that varied in the number of MCMC iterations (50,000 and 100,000) and the number of AFLP, microsatellite and candidate gene markers (575 and 927) for *K* = 1 to *K* = 10. The optimum number of K can be identified according to the maximum value of LnP(D) (Pritchard et al., 2000). In our data, the likelihood distribution increased slightly, leveled off and then decreased with a clear plateau from *K* = 3 to *K* = 5 (Figure S2A). Using the method of Evanno et al. (2005) in two independent runs with a total of 575 AFLP and SSR markers (burn-in 10,000; MCMC 50,000, and 100,000) and one run with 627 markers (burn-in 10,000; MCMC 50,000), the maximum for Δ*K* was estimated at *K* = 3 (Figure S2B). The result was confirmed by additional independent runs (data not shown).

**Figure 1:**
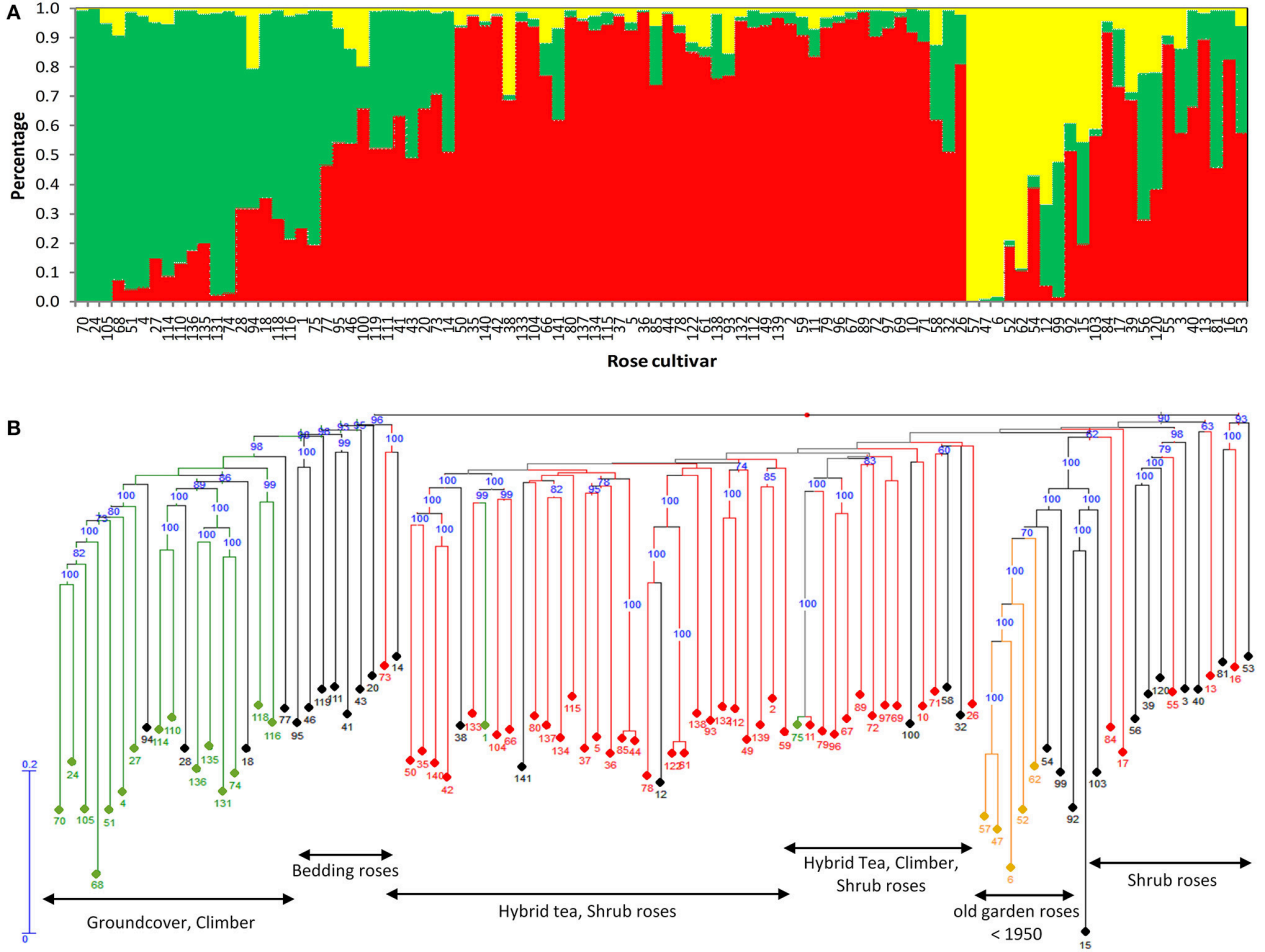
**Population structure of the 96 cultivars (A)** Bar plots of the proportion of membership of each cultivar to a subpopulation assigned for *K* = 3 using STRUCTURE 2.3.4. The numbering of each cultivar is displayed on the x-axis. Each subpopulation is indicated by a specific color. **(B)** Neighbor-joining tree of the association panel generated with DARwin 5.0.158 using 16,038 SNP markers. Members of subpopulation I are highlighted in red, subpopulation II in green and subpopulation III in yellow. Hybrid individuals (less than 0.7 of membership to any subpopulation) are represented in black. Each of the 96 cultivars is symbolized by its code number from 1 to 141 (Table S1). Bootstrap values (%) are given when greater than 70.

The structure of the population at *K* = 3 is visualized in Figure 1A. Below it are depicted the concordant results from the cluster analysis of the SNP data (Figure 1B). When using a threshold of 0.7 to assign individuals to a subpopulation or to classify them as a mix or as hybrid individuals (as (D'hoop et al., 2010) used in highly heterozygous tetraploid potato), subpopulation I, the largest group, consisted of 44 cultivars, which clustered according to their type or habit, particularly hybrid tea and floribunda roses. Subpopulation II contained 17 recently bred (1985–2011) cultivars, except for New Dawn (1930), which all have a groundcover habit. Subpopulation III, the smallest group, comprised only five cultivars belonging to the old garden type of roses: Damask (‘Rose de Resht’, before 1900), Alba (‘Small Maidens Blush’, 1797), Bourbon (‘Louise Odier’, 1851), Hybrid Perpetual (‘Mrs. John Laing’, 1885) and Portland (‘Mme Knorr’, 1855 und ‘Mme Boll’, 1858) roses. The positioning of the cultivars was supported by high bootstrap values, except for some in the first subpopulation. However, 30 cultivars could not be assigned to any of the three subpopulations using the threshold of 0.7 for the classification. The results of a principal coordinate analysis (PCoA; Figure S3) based on genetic distances agreed with this division in three subpopulations. The hybrid cultivars that shared part of subpopulations I and II can be observed as an intersection (black dots) between these subpopulations.

### Genetic diversity analysis

The kinship estimates based on SNPs (Figure S4) indicated no familial relationship between most of the rose genotypes. Approximately 59% of the pairwise kinship coefficients had values near zero (<0.005). Higher kinship estimates (0.10–0.20) were found between climber and ground cover roses. The highest values (0.26–0.39) were found within the group of old garden roses (population III).

The heterozygosity was determined based on the SNP data without considering the dosage of the markers (i.e., AAAB, AABB, and ABBB are all classified as heterozygote). When defined this way, the percentage of heterozygous loci is identical to the percentage polymorphic loci. On average, varieties displayed 55.2% of heterozygous loci ranging from 27% for variety No. 105 and 66.9% for variety No. 2. No correlation between heterozygosity and the age of the variety was observed (Figure S5).

**Figure 2:**
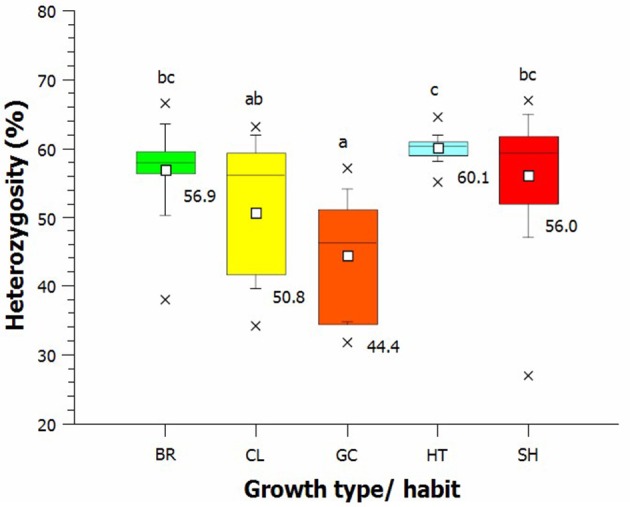
**Average heterozygosity of SNPs in different rose growth types (BR, bedding roses; CL, climber; GC, ground cover; HT, Hybrid tea; and SH, shrub roses)**. Small white square = mean; continuous line = median; asterisk = minimum, maximum; box = 1st and 3rd quartiles; and whisker = standard deviation.

On the other hand, there were significant differences between ground cover, climber, bedding roses, Hybrid teas and shrub roses, both in the average level of heterozygosity and in the variation in heterozygosity within groups (Figure 2). Groundcover roses had the lowest heterozygosity (44.4% heterozygous loci). Hybrid teas were significantly higher in heterozygosity (60.1%) compared to climbers and ground cover roses.

### Phenotypic characterization of morphological traits

#### Total anthocyanins

**Figure 3:**
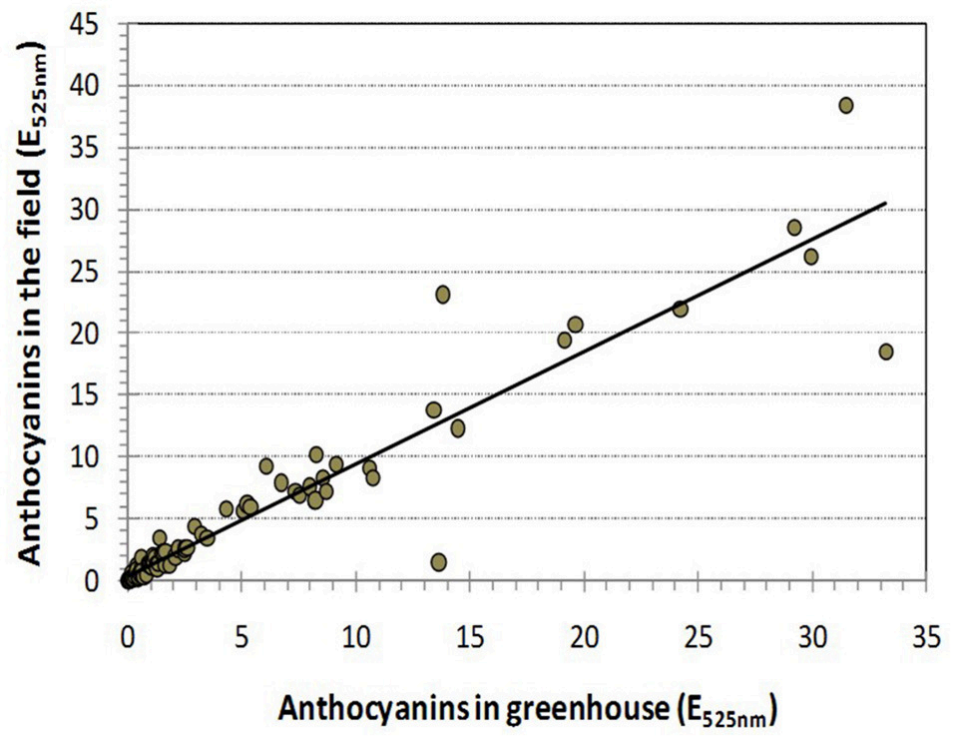
**Pearson's correlation between the total amount of anthocyanin that accumulated in rose cultivars grown in the field and in the greenhouse (*r* = 0.942)**.

In many cultivars, the anthocyanin content in the petals was low or not detectable, which was not unexpected because 23 cultivars had a white and yellow flower color. The measured anthocyanins were in the range from E_525nm_ = 0.35 to E_525nm_ = 33.22 in the greenhouse and from E_525nm_ = 0.33 to E_525nm_ = 38.39 in the field. The distribution was skewed to the left (Figure S6 and Table 1) and very similar for both environments (*r* = 0.942, Figure 3).

**Table 1:** **Descriptive statistics of the investigated traits**.

**Trait**	**Mean**	**Median**	**Stdev**	**Stderr**	**Variance**	**Max**	**Min**	**Skewness**	***N***
Anthocyanins (greenhouse)	4.78	1.19	7.639	0.784	58.35	33.22	0.035	2.261	95
Anthocyanins (field)	4.56	1.41	7.198	0.750	51.81	38.39	0.033	2.506	92
Carotenoids (greenhouse)	0.14	0.07	0.175	0.018	0.03	0.88	0.012	1.957	94

Stderr, standard error; Stdev, standard deviation; Max, maximum; min, minimum; N, number.

#### Carotenoids

Carotenoids in the rose petal extracts were measured by their characteristic absorbance at 442 nm (Figure S1) from all cultivars cultivated in the greenhouse at the Federal Plant Variety Office Hannover and from 20 of the cultivars in the field at Herrenhausen. Because the Spearman rank correlation between the measured values was very high (Spearman's rho = 0.939) the carotenoid contents of the additional 76 cultivars grown in the field were not estimated to avoid redundant data (Figure S8). In white flowers and in many red flowers, the yellowish to orange pigments were not present or only found as minor pigments in the petals. The maximum amount of carotenoids was detected in the yellow flowering cultivar “China Girl” (E_442nm_ = 0.8854). In roses classified as orange, a balanced occurrence of anthocyanins and carotenoids was always measured. However, there was no overall correlation between the anthocyanin and carotenoid content in the rose petals (*r* = −0.1803, *p* = 0.0836).

### Marker trait associations

#### Anthocyanins

Marker-trait associations were calculated in Tassel 3.0 with 39,831 SNPs based on their diploid allelic state (A:A, A:B, and B:B). The tetraploid dosage of the SNP was not used for calculation because TASSEL 3.0 can only handle haploid and diploid genotypic data (user manual for Tassel 3.0, Buckler Lab, Cornell University, 2011).

**Figure 4:**
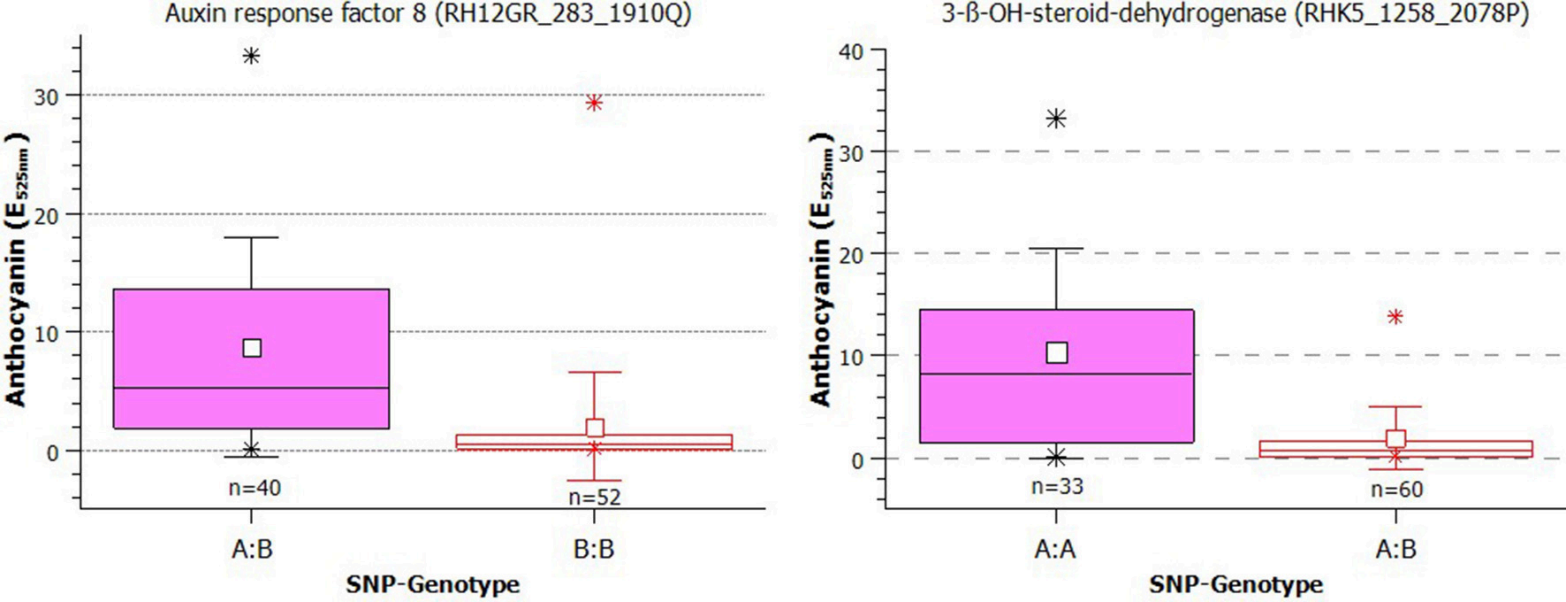
**Box plot of the effect of the SNPs in the auxin response factor 8 and 3-ß-OH-steroid-dehydrogenase genes on the anthocyanin content in rose petals (small white square = mean; continuous line = median; asterisk = minimum, maximum; box = 1st and 3rd quartiles; and whisker = standard deviation)**. Tassel compared only two genotype classes (one homozygote and one heterozygote for the SNP). The varieties were grouped according to their SNP type as A:A, A:B, or B:B. The influence of population structure and kinship were not considered. Note that the calculations were performed using transformed data, but the plots show the untransformed values.

The anthocyanin content in greenhouse-grown roses was significantly associated with 17 SNP markers, five of which were also associated with the anthocyanin content from field-grown roses (Table 2). These SNPs were Rh12GR_283_1910Q (in the Auxin response factor 8 gene), RhK5_1258_2078P (3 ß-OH-steroid-dehydrogenase/decarboxylase isoform 2), RhK5_7371_202Q (Glutathione S-transferase), Rh12GR_20064_1031P and RhMCRND_20203_163Q (both are in the Medium-chain-fatty-acid-CoA ligase). We estimated the effects of the SNPs on the anthocyanin content in greenhouse-grown roses from 3.985 to 7.589 (Table 2). Under both conditions, the largest effect was found for the marker RhK5_1258_2078P. In Figure 4, two boxplots of anthocyanin content showed the direct effects of the markers. For the SNP in the auxin response factor 8 gene, the mean for the heterozygous genotypes A:B was 8.63 (E_525nm_) and was significantly higher (*p* = 5.03E-8) than 1.93 (E_525nm_) for the homozygous B:B genotypes. For the 3-ß-OH-steroid-dehydrogenase SNP, the difference between the mean of the two groups (A:A = 10.26; A:B = 1.91) was also significant (*p* = 8.56E-7).

**Table 2:** **Significant SNPs for anthocyanin content in rose petals from greenhouse- and field-grown roses**.

**Greenhouse**	***p*-value**	**Effect^b^**				**Function (identified gene)**
**Combined^a^**		**(E_525nm_)**	**Allele A:A**	**Allele A:B**	**Allele B:B**	
Rh12GR_283_1910Q	2.35E-09	−5.412	−	77	104	gene31631-v1.0-hybrid_Auxin_response_factor_8_ (putative)
RhMCRND_20203_163Q	7.20E-09	−4.091	−	81	102	gene31669-v1.0-hybrid_Medium-chain-fatty-acid-CoA_ligase_(probable)
Rh12GR_20064_1031P	1.86E-07	−4.521	102	75	−	gene31669-v1.0-hybrid_Medium-chain-fatty-acid-CoA_ligase_(probable)
RhK5_7371_202Q	2.02E-07	−4.492	−	79	104	gene31672-v1.0-hybrid_Glutathione_S-transferase_ (similar_to)
RhK5_1258_2078P	3.75E-07	−6.674	66	117	−	gene08692-v1.0-hybrid_3beta-hydroxysteroid-dehydrogenase/decarboxylase_isoform_2_(At3BETAHSD/D2)_(similar_to)
RhMCRND_7128_1021Q	4.51E-07	−1.935	−	83	92	gene31668-v1.0-hybrid_Medium-chain-fatty-acid–CoA_ligase_(probable)
Rh12GR_64257_531Q	4.62E-07	−5.776	−	61	106	gene16750-v1.0-hybrid_hypothetical_protein
RhK5_2615_1401Q	5.58E-07	−5.549	−	76	105	gene09148-v1.0-hybrid_Cysteine_proteinase _RD21a_ (RD21),_Precursor_(putative)
RhK5_15799_993Q	6.00E-07	−7.015	−	59	106	gene26062-v1.0-hybrid_Formin-like_protein_20_(AtFH20)_(similar_to)
RhMCRND_982_2342Q	1.06E-06	−5.538	−	60	117	gene26062-v1.0-hybrid_Formin-like_protein_20_ (AtFH20)_(similar_to)
RhK5_1553_678P	1.10E-06	−4.719	119	65	−	gene26062-v1.0-hybrid_Formin-like_protein_20_ (AtFH20)_(similar_to)
RhK5_9221_621Q	1.11E-06	6.179	76	85	−	gene16819-v1.0-hybrid_Thioredoxin_F-type_2, _chloroplastic_(Trx-F2),_Precursor_(similar_to)
Rh12GR_77973_217Q	1.17E-06	−4.794	108	75	−	−
RhK5_12663_103Q	1.32E-06	−3.628	−	69	114	gene24536-v1.0-hybrid_Possible_hemolysin_C_ (probable)
Rh12GR_17814_425Q	1.62E-06	−3.136	104	81	−	gene31679-v1.0-hybrid_Ubiquitin-like_protein_SMT3 _(probable)
RhK5_15799_993P	1.80E-06	−5.472	−	60	119	gene26062-v1.0-hybrid_Formin-like_protein_20_ (AtFH20)_(similar_to)
RhMCRND_1369_1182Q	2.00E-06	5.215		62	121	gene26062-v1.0-hybrid_Formin-like_protein_20_(AtFH20)_(similar_to)
Rh12GR_283_1910P	2.38E-06	3.516	−	81	94	gene31631-v1.0-hybrid_Auxin_response_factor_8_ (putative)
RhK5_19460_153P	2.45E-06	4.855	123	61	−	gene04292-v1.0-hybrid_Cytokinin-O-glucosyltransferase_2_(AtZOG2)_(probable)
Rh12GR_38264_410P	2.58E-06	−4.584	88	79	−	−
**Greenhouse**	***p*****-value**	**Effect^b^ (E_525nm_)**	**Allele A:A**	**Allele A:B**	**Allele B:B**	**Function (identified gene)**
Rh12GR_283_1910Q	9.73E-11	−5.740	−	40	52	gene31631-v1.0-hybrid_Auxin_response_factor_8_ (putative)
RhK5_1439_806P	8.10E-09	−4.927	68	19	−	U3 small nucleolar RNA-associated protein 4/UTP4
RhMCRND_20203_163Q	1.17E-08	−5.135	−	42	51	gene31669-v1.0-hybrid_Medium-chain-fatty-acid–CoA_ligase_(probable)
RhK5_1258_2078P	9.44E-08	−7.589	33	60	−	gene08692-v1.0-hybrid_3beta-hydroxysteroid-dehydrogenase/decarboxylase_isoform_2_(At3BETAHSD/D2)_(similar_to)
Rh12GR_20064_1031P	1.21E-07	−5.112	51	39	−	gene31669-v1.0-hybrid_Medium-chain-fatty-acid–CoA_ligase_(probable)
RhK5_7371_202Q	1.23E-07	−5.426	−	41	52	gene31672-v1.0-hybrid_Glutathione_S-transferase_ (similar_to)
RhMCRND_319_1197P	7.49E-07	4.398	−	60	26	Urease_(similar_to)
RhK5_12076_566Q	7.74E-07	−5.193	−	42	52	Photosystem_I_reaction_center_subunit_XI,_ chloroplastic_(PSI-L),_Precursor_
RhMCRND_982_2342Q	8.43E-07	−6.928	−	30	60	gene26062-v1.0-hybrid_Formin-like_protein_20_ (AtFH20)_(similar_to)
Rh12GR_17814_425Q	8.45E-07	−4.391	52	42	−	gene31679-v1.0-hybrid_Ubiquitin-like_protein_SMT3 _(probable)
Rh12GR_92431_4144Q	8.48E-07	−3.985	−	35	58	gene31646-v1.0-hybrid_Serine/threonine-protein_ kinase_PBS1_(probable)
RhK5_2615_1401Q	8.64E-07	−5.525	−	39	53	gene09148-v1.0-hybrid_Cysteine_proteinase_ RD21a_(RD21),_Precursor_(putative)
RhK5_15799_993P	1.00E-06	−6.814	-	30	61	gene26062-v1.0-hybrid_Formin-like_protein_20_ (AtFH20)_(similar_to)
RhK5_5774_854P	1.11E-06	4.321	−	62	26	Translation_initiation_factor_IF-2_
RhK5_12663_103Q	1.37E-06	4.258	−	33	57	gene24536-v1.0-hybrid_Possible_hemolysin_C _(probable)
RhK5_21626_409P	1.66E-06	4.064	−	61	25	gene07023-v1.0-hybrid_Anthranilate_ phosphoribosyltransferase_(probable)
Rh12GR_3292_1365P	2.20E-06	4.122	18	68	−	gene10566-v1.0-hybrid_Putative_indole-3-acetic_acid-amido_synthetase_GH3.9_(AtGH3-9)
**Field**	***p*****-value**	**Effect^b^ (E_525nm_)**	**Allele A:A**	**Allele A:B**	**Allele B:B**	**Function (identified gene)**
Rh12GR_283_1910Q	4.51E-08	−5.451	−	37	52	gene31631-v1.0-hybrid_Auxin_response_factor_8_ (putative)
RhMCRND_20203_163Q	5.29E-08	−2.631	−	40	51	gene31669-v1.0-hybrid_ Medium-chain-fatty-acid–CoA_ligase_(probable)
RhK5_1258_2078P	1.30E-07	−6.511	33	57	−	gene08692-v1.0-hybrid_3beta-hydroxysteroid-dehydrogenase/decarboxylase_isoform_2_(At3BETAHSD/D2)_(similar_to)
Rh12GR_20064_1031P	1.82E-06	5.051	51	36	−	gene31669-v1.0-hybrid_Medium-chain-fatty-acid–CoA_ligase_(probable)
RhK5_7371_202Q	1.94E-06	5.100	−	38	52	gene31672-v1.0-hybrid_Glutathione_S-transferase_ (similar_to)
RhMCRND_7128_1021Q	2.37E-06	−1.901	−	39	48	gene31668-v1.0-hybrid_Medium-chain-fatty-acid–CoA_ligase_(probable)

a Total number of A:A, A:B, and B:B genotypes was counted in two environments and exceeded the number of 96 cultivars.

b The effect was presented as untransformed values.

Because the genome sequence of *Rosa sp*. is not complete, the contigs of 133 SNPs with hits just below the Bonferroni threshold plus the 17 significant SNPs were blasted against the closely related genomes of *Fragaria vesca* and *Prunus persica* and mapped on these genomes (**Figures 6A,B**, Table S6). The assumption is that both genomes display sufficient microsynteny to the rose genome. This assumption is supported by the fact that these rose SNPs clustered in distinct regions of the *Fragaria* genome, particularly in linkage groups Fvb1, Fvb2, Fvb4, Fvb5, and Fvb6, and in the partly homologous linkage groups Pp01, Pp03, Pp05, Pp06, and Pp08 of *Prunus persica* (https://www.rosaceae.org/gb/gbrowse_syn/peach_apple_strawberry/). A blast of the contigs located three of the SNP markers in the coding region of anthocyanin biosynthesis genes, 4-coumarate-ligase (4_CL), flavonoid 3′-hydroxylase (F3′H) and glutathione-S-transferase (GST). The positions of these genes of the anthocyanin biosynthesis pathway and of further transcription factors are shown in the genome plots as green dots (**Figures 6A,B**). The putative transcription factors that are associated with anthocyanin accumulation include a ubiquitin-like protein SMT3 (SUMO1), WRKY transcription factor 17 and UTP4/Cirhin, a WD40 repeat protein (Freed et al., 2012).

#### Carotenoids

Because as many as 351 SNPs were significantly associated with the accumulation of carotenoids in rose petals and surpassed the Bonferroni threshold of α = 2.62e-6 (Table S7), the effects of the significant SNPs on carotenoid content ranged from 0.00015 to 0.259 (E_442nm_). Most of the significant SNPs formed two large clusters in linkage group 5 of *F. vesca* and *P. persica* with more than 250 SNPs (**Figures 7A,B**). They may be located on a part of the chromosome with low recombination. Two of the SNPs were located on contigs encoding genes of the MEP (methylerythritol 4-phosphate) pathway, CMS (2-c-methyl-d-erythritol-cyclodiphosphatase) and DXR (1-deoxy-d-xylulose 5-phosphate reducto-isomerase). A third enzyme, more upstream in the carotenoid biosynthesis, is Zeaxanthin epoxidase (ZEP, *p* = 8.77e-6). It was located in linkage groups Fvb1 and Pp07. ZEP is a part of the branch of the ß-carotenoid biosynthesis that catalyzes the step from zeaxanthin to violaxanthin. Additionally, several significant SNPs were mapped to linkage group four of *F. vesca* and to linkage group one of *P. persica*. The positions of these SNPs were close to the assumed position of a carotenoid cleavage dioxygenase gene, CCD4. Other significant SNPs were located in two cytochrome P450 monooxygenases (CP450): cytochrome P450_71A24 (Pp04, Fvb5) and cytochrome CP450_CYP749A22 (Pp01, Fvb4).

**Figure 5:**
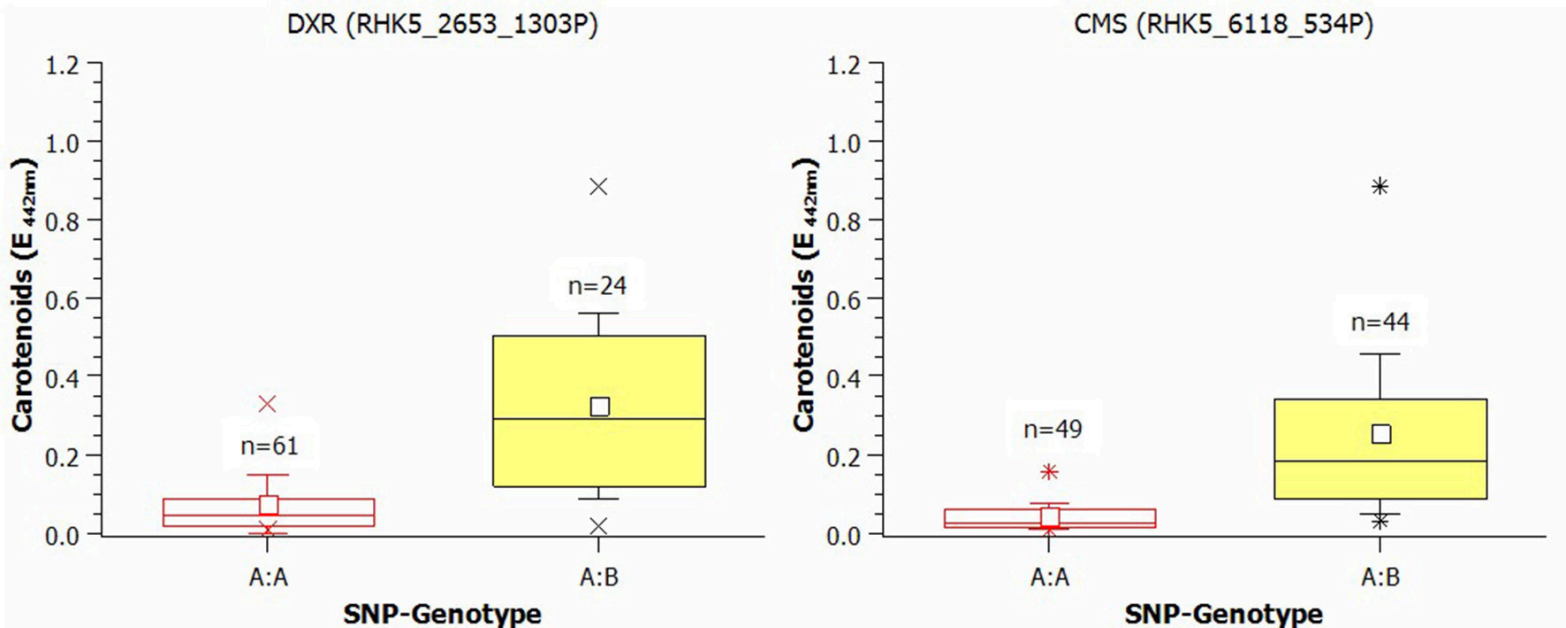
**Box plot of the effect of SNPs in 1-Deoxy-D-xylulose-5-phosphate-reductoisomerase (DXR) and in 4-Diphospho-cytidyl-2-C-methyl-d-erythritol-synthase (CMS) on the carotenoid content in rose petals (small white square = mean; continuous line = median; asterisk = minimum, maximum; box = 1st and 3rd quartiles; and whisker = standard deviation)**. The varieties were grouped according to their SNP type as A:B or B:B. The influence of population structure and kinship was not included. The averages were calculated after transformation but are presented as untransformed values.

The effects of SNPs on the carotenoid biosynthesis genes CMS and DXR are shown as box plots in Figure 5. For the DXR-SNP, the mean of the homozygous A:A genotypes was 0.074 (E_442nm_), whereas the mean for the A:B genotypes was 0.325 (E_442nm_). The effect of the SNP for CMS, the subsequent gene after DXR in the carotenoid pathway, was 0.206 (E_442nm_), and the mean was also obviously higher in the A:B group [A:A = 0.0475 (E_442nm_); A:B = 0.253 (E_442nm_)].

## Discussion

Floral traits in ornamental roses are determined by a number of quantitative traits, e.g., petal number, flower size and a large number of secondary metabolites that constitute flower color and flower fragrance. Here, we applied GWAS based on the rose WagRhSNP array to analyse factors influencing the amount of anthocyanins and carotenoids in petals. In this study, we tried for the first time to utilize association genetics to exploit the vast phenotypic variation in mainly tetraploid cultivated roses for an analysis of quantitative traits.

### Heterozygosity is not influenced by cultivar age

The large number of markers used to genotype the association panel revealed a high average heterozygosity of 55.2% in the population, which is in agreement with previous studies on marker diversity in cultivated roses (Debener et al., 1996; Esselink et al., 2003). The value of heterozygosity is in fact the percentage of polymorphic SNPs; thus, it is not surprising that the value is somewhat lower than that found with 24 SSR markers in garden roses (Vukosavljev et al., 2013). Unlike many other cultivated plant species (Kilian et al., 2007; Gil-Ariza et al., 2009; Wang et al., 2010; Gross et al., 2014), we did not see a reduction over time when we considered the year of release of the variety. This may corroborate observations on the sensitivity of rose for inbreeding depression, which makes selfing unsuitable as a breeding strategy (Pipino et al., 2011). It might also be the result of the large range of ornamental traits that breeders have selected for, causing selection to never be consistently in the same direction. We found a lower percentage of polymorphic loci in the climber (40.9%) and ground cover genotypes (50.2%). This could indicate some degree of ascertainment bias if the genetic background of these groups of roses is partly different from those of the cut and garden roses from which the SNPs derived (Koning-Boucoiran et al., 2015; Smulders et al., 2015). This would require further studies, for instance, a study that searches for homologous regions in the genomes of diploid species that have contributed to the tetraploid groups.

### Population structure

Population structure and relatedness between genotypes can be confounding factors in association mapping (Nordborg and Weigel, 2008). Minimal population structure or relatedness will result in high statistical power, but larger collections offer more power, and a collection of 100–500 individuals is recommended (Hirschhorn and Daly, 2005; Rafalski, 2010). The STRUCTURE software with the implemented Bayesian clustering approach is a common tool to assess population structure with a moderate number of markers. Together with the estimation of kinship, this tool can reduce the rate of false positives in association mapping (Pritchard et al., 2000; Rafalski, 2010). With 400 AFLP and 175 SSR markers, we identified three subpopulations in our rose association panel of 96 different cultivars. This is comparable to the sample sizes used in various association mapping studies with other crop species. For instance, the association panel of Lindqvist-Kreuze et al. (2014) comprised 103 potato genotypes, and population structure was estimated with 120 SNP markers. Seventy-one almond cultivars were the basis of an association study on kernel phytosterol content (Font i Forcada et al., 2015) in which population structure was corrected using 40 SSRs. Simko et al. (2009) used 68 lettuce cultivars for association with disease resistance and validated the detected marker trait association in a second set of 132 cultivars.

#### Using synteny

**Figure 6:**
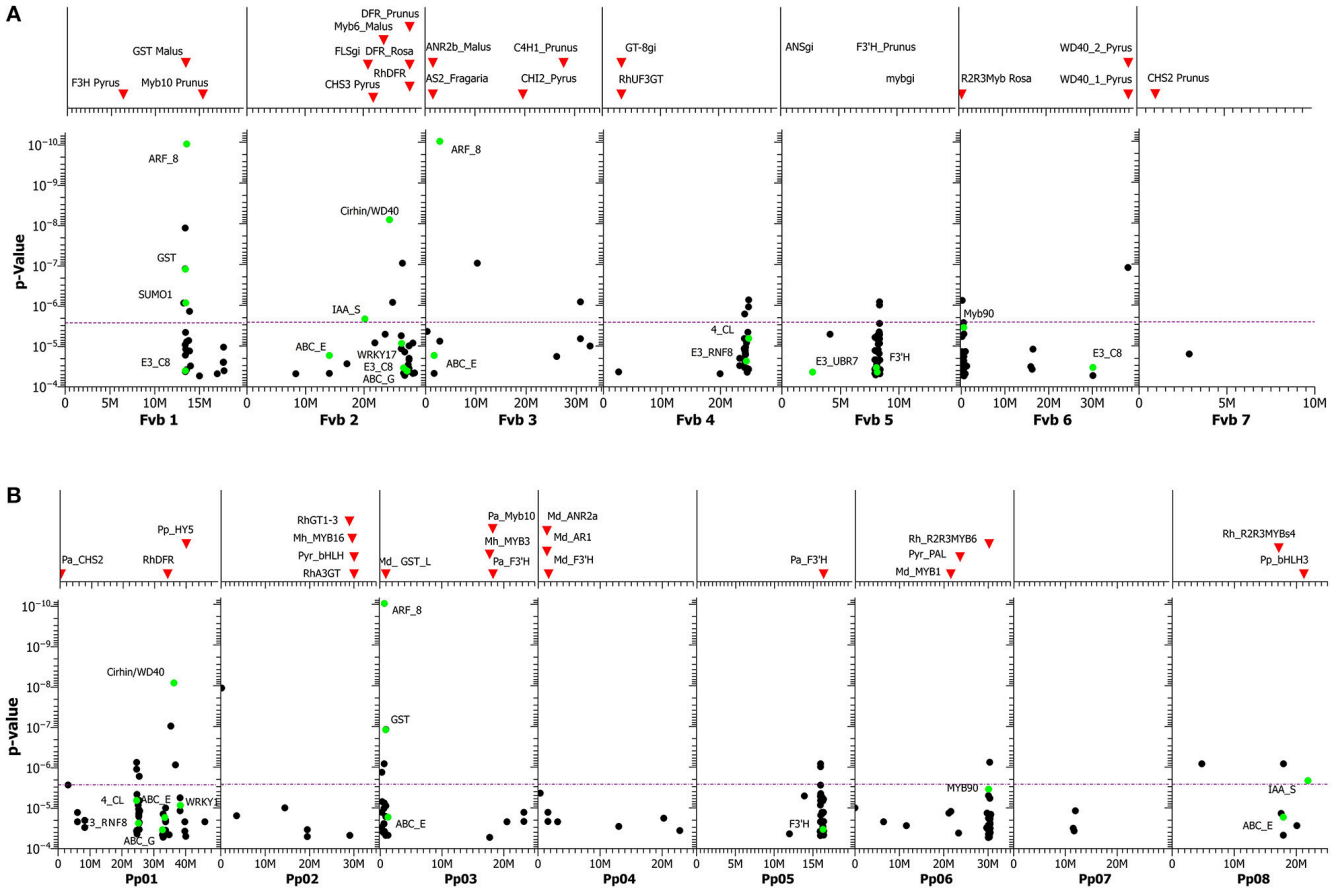
**(A)** GWAS for anthocyanin content. SNPs are mapped to the genome of homologous sequences in *Fragaria vesca*, including those in annotated genes (green dots). On top of the graph, the positions of various known candidate genes in the *F. vesca* genome sequence are shown as red triangles. The purple dotted line represents the Bonferroni adjusted significance level. For abbreviations of genes, including functions, see Table S8. **(B)** GWAS for anthocyanin content. SNPs are mapped to the genome of homologous sequences in *Prunus persica*, including those in annotated genes (green dots). On top of the graph, the positions of various known candidate genes in the *P. persica* genome sequence are shown as red triangles. The purple dotted line represents the Bonferroni adjusted significance level. For abbreviations of genes, including functions, see Table S8.

A major problem of association studies is filtering true marker-trait associations out of a large number of false-positive associations. Next to *p*-values and the extent of the observed effects, the clustering of significant markers in particular genomic regions is a criterion for true marker trait associations. However, for rose, no completed genome sequence is available and relative positions are known only for a small subset of the markers that we applied in this study. Therefore, we used two related rosaceous genomes assuming sufficient microsynteny to the rose genome: the very closely related genome of strawberry and the peach genome, which represents the next-closest relative (Shulaev et al., 2008). In several conventional marker mapping studies, strawberry was shown to be highly similar in its genome structure and marker order with only minor differences to roses (Gar et al., 2011; Spiller et al., 2011; Terefe-Ayana et al., 2012). This strategy proved informative, as we found a couple of clear clusters of significantly associated SNPs in both genomes. Our attempt to locate candidate markers to a genomic region failed for some markers (e.g., SUMO1) in one of the two genomes, but many others were located in both genomes (Figures 6A,B).

### SNPs associated with anthocyanin accumulation in rose petals

As a simple measure for red and pink flower colors, we used the total amounts of anthocyanins determined by spectrophotometry in extracts prepared at defined flower stages. This has the advantage that other factors, e.g., cellular pH, cofactors, or flower age, which influence the visual characteristics of the anthocyanins in the natural context, are excluded; therefore, the phenotypic complexity can be partially reduced. This strategy was successful, as evidenced by the high correlation between the greenhouse and the field environment, which differ significantly in terms of temperature profiles and UV radiation.

Our study indicates that at least five genomic regions contain factors influencing anthocyanin concentration. Interestingly, all of these regions contained either SNP markers from genes with known functions in anthocyanin metabolism or candidate genes mapping to these regions (Table S3). The cluster in linkage group Fvb1 of *Fragaria* comprised the marker with the lowest *p*-value, a homolog to an auxin response factor known to influence anthocyanin concentration by regulating auxin expression in apple (Ji et al., 2015), Arabidopsis (Liu et al., 2014), tobacco (Zhu et al., 2013) and cabbage (Kang and Burg, 1973), and a family member of GSTs, which have important functions in anthocyanin transport from the cytosol to the vacuole. GSTs are responsible for color variation in a number of ornamental species, including petunia and carnation (Zhao, 2015). Homologues of the SUMO-1 transcription factor on *Fragaria* chromosome 1 are regulators of signal transduction in auxin signaling in plants (del Pozo et al., 1998; Vierstra and Callis, 1999). Because chromosome 1 of *Fragaria* is mostly collinear with the ICM linkage group 2 of *Rosa* (Gar et al., 2011), this region is likely to be close to the QTL for anthocyanin content in a diploid biparental rose population (Henz et al., 2015).

Several transcription factors on *Fragaria* chromosome 2 were associated with anthocyanin accumulation: WRKY transcription factor 17, a ubiquitin-like_protein_SMT3 (SUMO1), and UTP4/Cirhin, a WD40 repeat protein (Freed et al., 2012). Zorrilla-Fontanesi et al. (2011) detected in strawberry associations between a putative R2R3 Myb transcription factor and QTLs for anthocyanin accumulation in linkage group 2. The *Fragaria* chromosome 2 is largely syntenic with rose the ICM linkage group 6 harboring major QTLs for the anthocyanin content, which were also stable across several environments (Henz et al., 2015). The cluster of SNPs mapped on *Fragaria* chromosome 4 included SNPs in the rose 4_CL gene and various Myb transcription factors, which are known to regulate anthocyanin biosynthesis. The transcription factor myb90-like (myb90), also known as “Production of Anthocyanin Pigment 2” (PAP2), was identified on *Fragaria* chromosome 6 and is a member of the MBW-complex (Maier and Hoecker, 2015). The MBW-complex activates anthocyanin biosynthetic genes and is a complex of the transcription factors R2R3-Myb, basic Helix-Loop-Helix (bHLH), and WD40 proteins (Petroni and Tonelli, 2011; Maier and Hoecker, 2015).

### SNPs associated with carotenoid accumulation in rose petals

Eugster and Märki-Fischer (1991) could identify nearly 40 different carotenoids in the extract of rose petals, most prominently Violaxanthin together with Auroxanthin, Luteoxanthin and ß-Carotene (Ohmiya, 2011). Glick (2009) identified as main components Violaxanthin and Neoxanthin, which comprised 85% of the total carotenoid content in the petals of the rose cultivar “Frisco.” The biosynthetic pathway to Violaxanthin occurs via Zeaxanthin, and the modification is catalyzed by the enzyme Zeaxanthin epoxidase (ZEP). The carotenoids in the rose petals in our association study panel were characterized spectroscopically at 442 nm, which does not distinguish between different carotenoids. We detected as many as 303 significant SNPs associated with carotenoid content, 250 of which clustered in two positions on chromosome 5 of both *Prunus* and *Fragaria*. The majority of these 250 significant SNPs are not located in the genes causing the effect themselves, but they are closely linked to one or few of such genes located on the same chromosomal regions with low recombination rates. This extreme clustering was most probably due to the high linkage disequilibrium around the two clusters. The causes of this LD are not clear yet. Possible reasons might be either linkage to factors suppressing recombination in roses or the presence of genes under high selection pressure in cultivated roses. However, the fact that these two large clusters of significant SNPs were detected on both the Fragaria and the Prunus genome independently indicates that this is not due to a computational artifact of the GWAS or potential assembly errors in the target genome regions, but a real effect of the chromosomal region. Both target sequences have been independently assembled by different research groups and our results on rose sequences that match the same region in both heterologous genomes indicate synteny for the location of these sequences in all three genomes. Due to the huge number of significant carotenoid SNPs, we further discuss only SNPs in potential candidate genes (Table S4).

Carotenoids are synthesized from isopentenyl diphosphate (IPP) and dimethylallyl diphosphate (DMAPP) via the MEP pathway. Enzymes of the MEP pathway significantly influence carotenoid production (Lois, 2000; Moehs et al., 2001; Rodríguez-Concepción et al., 2001, 2003; Carretero-Paulet et al., 2002, 2006). We identified two SNPs for carotenoid accumulation in roses in the coding region of genes of the MEP pathway: CMS and DXR. The importance of DXR was shown with *Arabidopsis*, as down-regulation resulted in reduced pigmentation and defects in chloroplast development, whereas overexpression led to the accumulation of isoprenoids, such as chlorophylls and carotenoids (Carretero-Paulet et al., 2006).

**Figure 7:**
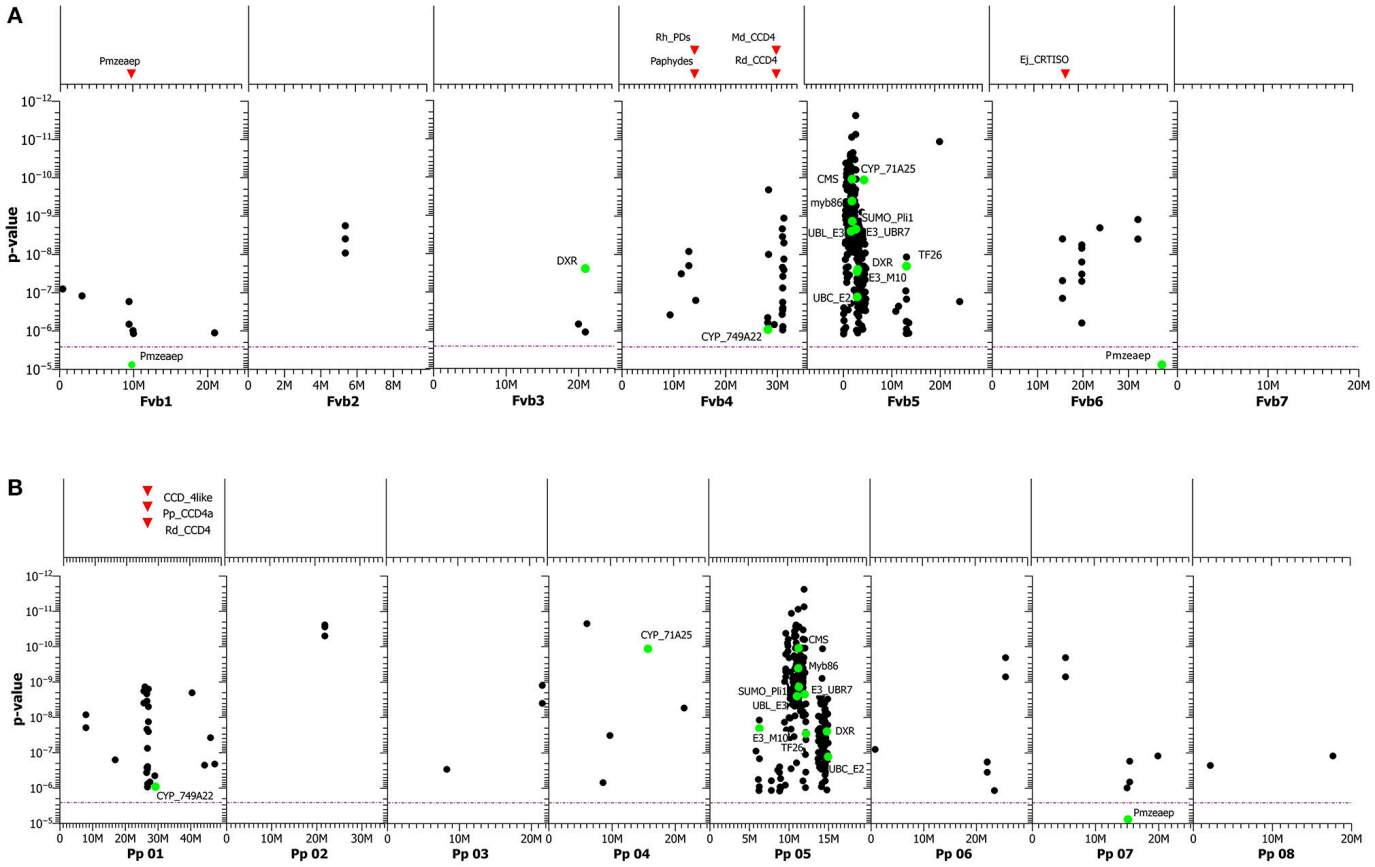
**(A)** GWAS of carotenoid content. SNPs are mapped to the genome of homologous sequences in *Fragaria vesca*, including those in annotated genes (green dots). On top of the graph, the positions of various known candidate genes in the *F. vesca* genome sequence are shown as red triangles. The purple dotted line represents the Bonferroni adjusted significance level. For abbreviations of genes, including functions, see Table S8. **(B)** GWAS of carotenoid content. SNPs are mapped to the genome of homologous sequences in *Prunus persica*, including those in annotated genes (green dots). On top of the graph, the positions of various known candidate genes in the *P. persica* genome sequence are shown as red triangles. The purple dotted line represents the Bonferroni adjusted significance level. For abbreviations of genes, including functions, see Table S8.

Carotenoid accumulation is also influenced by degradation. Campbell et al. (2010) showed that a reduced expression of the carotenoid cleavage dioxygenase 4 (CCD4) gene increased the carotenoid level in mature potato tubers 2- to 5-fold. Similarly, Glick (2009) found a high correlation between carotenoid degradation in the rose cultivars “Frisco” and “Golden gate” and the expression of RhCCD4. In chrysanthemum, the loss of the CmCCD4a gene caused a change in petal color from white to yellow; the level of color mutation from white to yellow may depend on the copy number of the CmCCD4a gene (Ohmiya et al., 2012; Yoshioka et al., 2012). We detected several significant SNPs for carotenoid accumulation on *Fragaria* chromosome 4 and *Prunus* chromosome 1, close to a CCD4 gene (Figures 7A,B, Table S5). It is obvious that the activity of the CCD4 genes affects the carotenoid content in different plants, but the role of the CCD4 genes in the degradation of carotenoids in roses needs more research.

Beside these SNPs located in the two regions on *Fragaria* chromosomes 4 and 5 and the corresponding *Prunus* chromosomes 1 and 5, we detected a SNP in the coding region of ZEP with a *p*-value of 8.77e-6 for the association located on *Fragaria* chromosome 1.

## Conclusions

This is the first association mapping study in rose. We focussed on the anthocyanin and carotenoid contents, which largely determine petal color. The phenotype data were collected in the field and in the greenhouse, and the overall levels of these compounds were not influenced by the differences in environment. To analyse the GWAS-associated SNPs in the absence of a rose genome sequence, we mapped the underlying rose contigs to the genome sequence of the related species *Fragaria vesca* and *Prunus persica*. Clusters of hits on these sequenced genomes in regions with known candidate genes confirmed that these genomes are probably largely syntenic and suggested that we identified 17 (anthocyanins) to 351 (carotenoids) trait-marker associations. Some of these had large effect sizes: these QTLs may be useful in breeding for intense flower colors in that parental breeding lines with combinations of several markers with high SNP dosages (duplex to quadruplex) might now be selected using the validated SNPs.

## Author contributions

DS conducted the experiments, did the statistical analysis and wrote most parts of the manuscript. RS conducted parts of the experiments. RV conducted part of the data analysis and contributed in writing the manuscript. MS contributed some of the data and contributed in writing the manuscript. ML contributed to the experimental setup, contributed data to some of the experiments and contributed in writing the manuscript. TD was involved in planning the experiments, in statistical analysis, and wrote parts of the manuscript.

## Funding

The research was in part funded within the program “Zentrales Innovationsprgramm Mittelstand (ZIM) of the German Bundesministerium für Wirtschaft und Energie (BMWi).”

### Conflict of interest statement

The authors declare that the research was conducted in the absence of any commercial or financial relationships that could be construed as a potential conflict of interest.

## Supplementary material

Figure S1 **Frequency distribution of carotenoids (A)** in greenhouse (Federal Plant Variety Office, Hannover) and absorption spectra of carotenoid extracts of three rose cultivars with the main carotenoid peak at 442 nm **(B)**.

Figure S2 **Estimation of the number of subpopulations. (A)** The mean log likelihood of data L(K) (±SD) (y-axis) as a function of K (x-axis) over ten repetitions for three independent runs in Structure 2.3.4. **(B)** Δ*K* (y-axis) as a function of K (mean ± SD) (x-axis) estimated with the method of Evanno et al. (2005) for the same runs.

Figure S3 **Principal component analysis of the association panel generated in DARwin 5.0.158 using 926 filtered AFLP and SSR markers**. The defined subpopulation I is circled in red, subpopulation II in green and subpopulation III in yellow. The cultivars are colored according to their share of belonging to a subpopulation (≥70%) as in the neighbor joining tree (Figure 1B).

Figure S4 **Distribution of relatedness in the association panel estimated in SPAGeDi**.

Figure S5 **Heterozygosity of the 96 rose varieties of the association panel plotted against the age of the varieties**.

Figure S6 **Frequency distribution of the total amount of anthocyanins in the greenhouse at the Federal Plant Variety Office (left) and in the field at Herrenhausen (right)**.

Figure S7 **Extracts from rose petals containing anthocyanins and carotenoids (above) and rose leaves containing carotenoids only (below)**.

Figure S8 **Spearman rank correlation between the total amount of carotenoids in rose petals from 20 cultivars grown in the field and in the greenhouse (Spearman's rho = 0.939)**.

Table S1 **Rose Association panel: Cultivars, breeder, origin, and breeding year of roses, code number (1–141) and flower color**. Cultivars with ploidy levels differing from tetraploid are labeled as ^(1)^ or ^(2)^.

Table S2 **Primer sequences, annealing temperature and linkage map location in rose (according to Spiller et al. 2011) of 27 SSR markers used in the association studies with the 96 rose cultivars**. Rh primers are from Esselink et al. (2003); RMS primers are published at http://www.wipo.int/pctdb/en/wo.jsp?wo=2003097869&IA=WO2003097869&DISPLAY=STATUS.

Table S3 **Candidate genes for anthocyanin biosynthesis localized in the genomes of *F. vesca* and *P. persica***.

Table S4 **Candidate genes for carotenoid biosysnthesis in the genomes of *F. vesca* and *P. persica***.

Table S5 **SNPs significantly associated with the anthocyanin and carotenoid contents after Bonferroni correction at a significance level of α = 2.62E-6**.

Table S6 **Anthocyanin SNP markers and their position on the genome of *Fragaria vesca* 2.0**.

Table S7 **SNPs significantly associated with carotenoid content and their function, *p*-value, and effect (untransformed value) on carotenoid content in rose petals**.

Table S8 **Abbreviations for known transcription factors, carotenoid and anthocyanin biosynthetic pathway genes**.
