# Supplementary figures and images for: Genome-Wide Association Analysis of the Anthocyanin and Carotenoid Contents of Rose Petals
Source: Front Plant Sci. 2016 Dec 6;7:1798. doi: 10.3389/fpls.2016.01798 (PMC5138216; doi:10.3389/fpls.2016.01798)

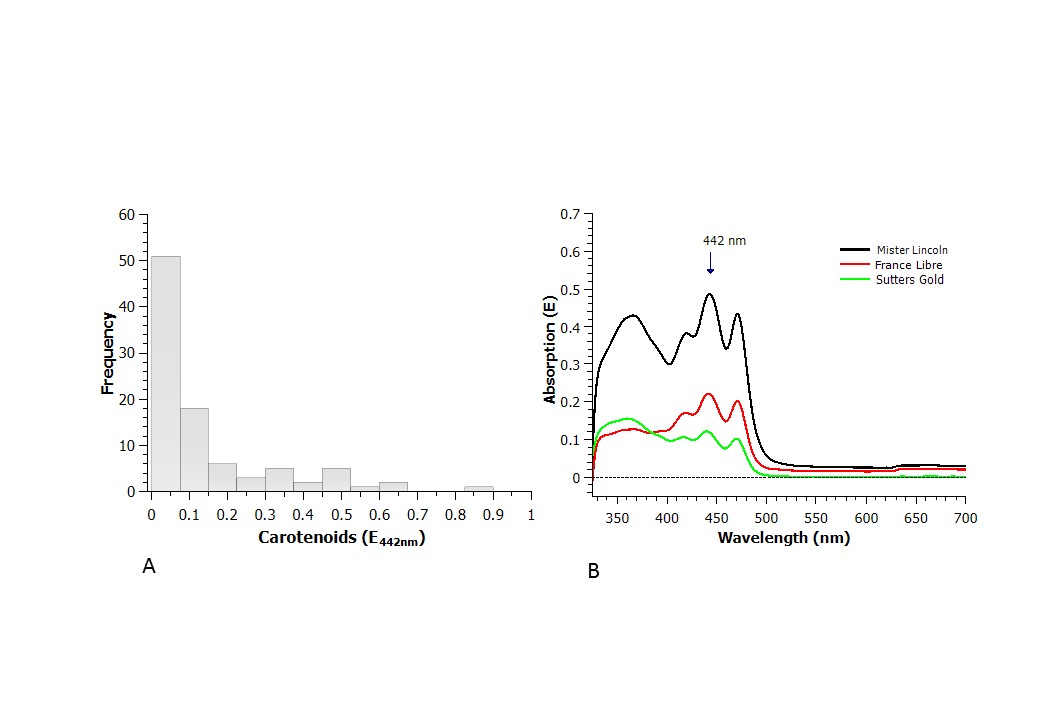

Supplement: Figure S1 — Frequency distribution of carotenoids (A) in greenhouse (Federal Plant Variety Office, Hannover) and absorption spectra of carotenoid extracts of three rose cultivars with the main carotenoid peak at 442 nm (B). [file Image1.JPEG]

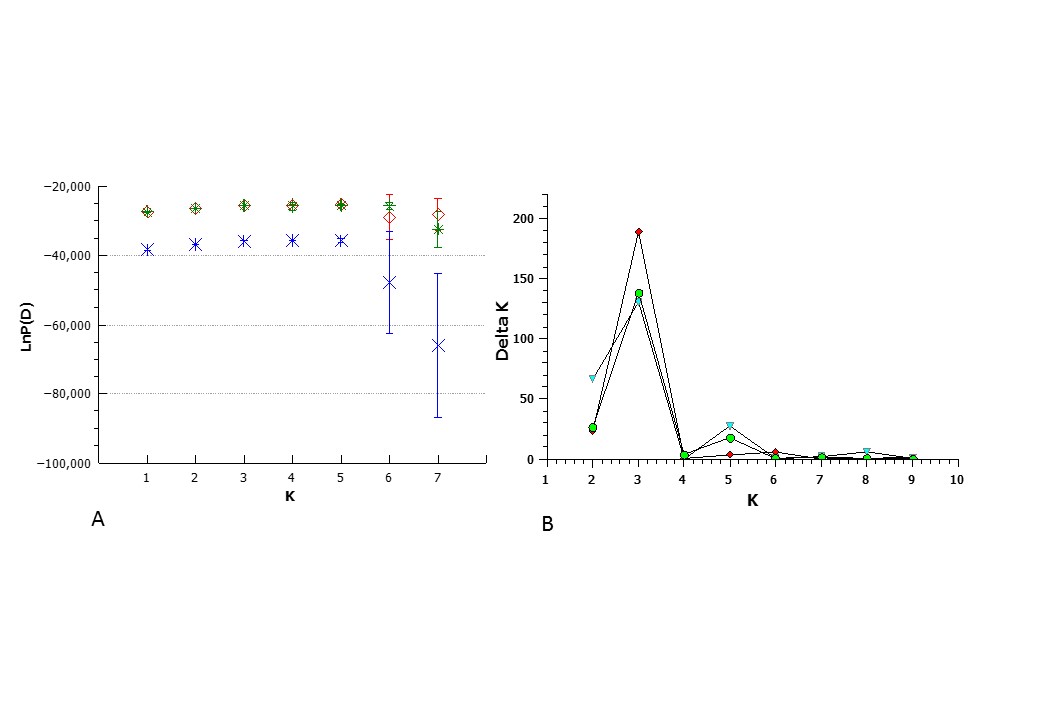

Supplement: Figure S2 — Estimation of the number of subpopulations. (A) The mean log likelihood of data L(K) (±SD) (y-axis) as a function of K (x-axis) over ten repetitions for three independent runs in Structure 2.3.4. (B) ΔK (y-axis) as a function of K (mean ± SD) (x-axis) estimated with the method of Evanno et al. (2005) for the same runs. [file Image2.JPEG]

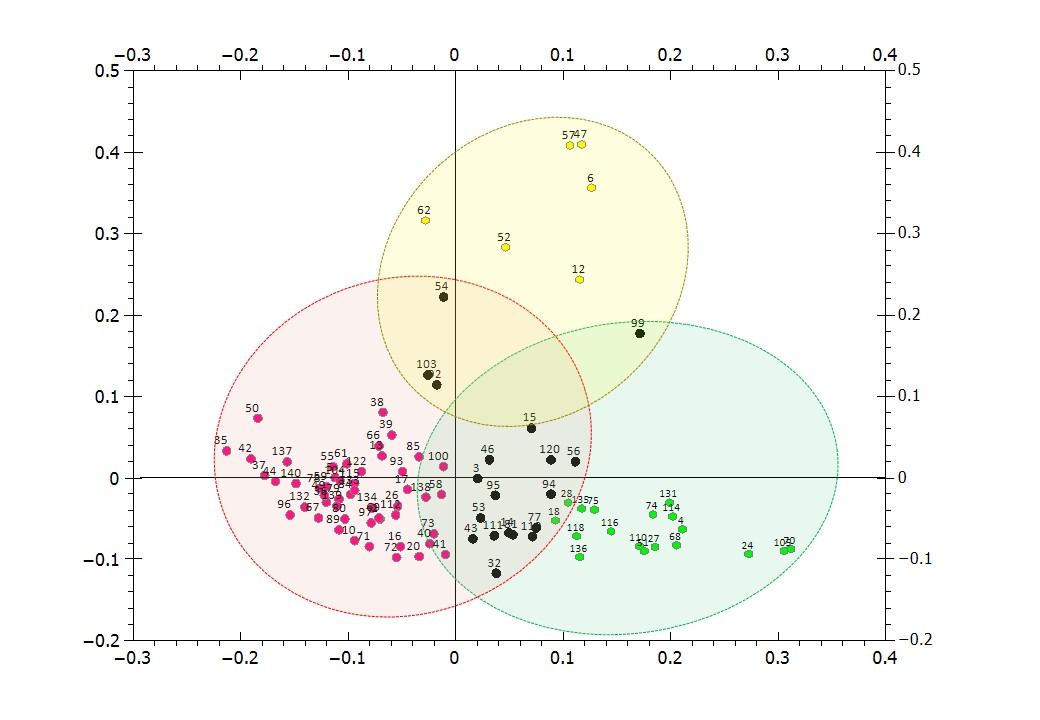

Supplement: Figure S3 — Principal component analysis of the association panel generated in DARwin 5.0.158 using 926 filtered AFLP and SSR markers. The defined subpopulation I is circled in red, subpopulation II in green and subpopulation III in yellow. The cultivars are colored according to their share of belonging to a subpopulation (≥70%) as in the neighbor joining tree (Figure 1B). [file Image3.JPEG]

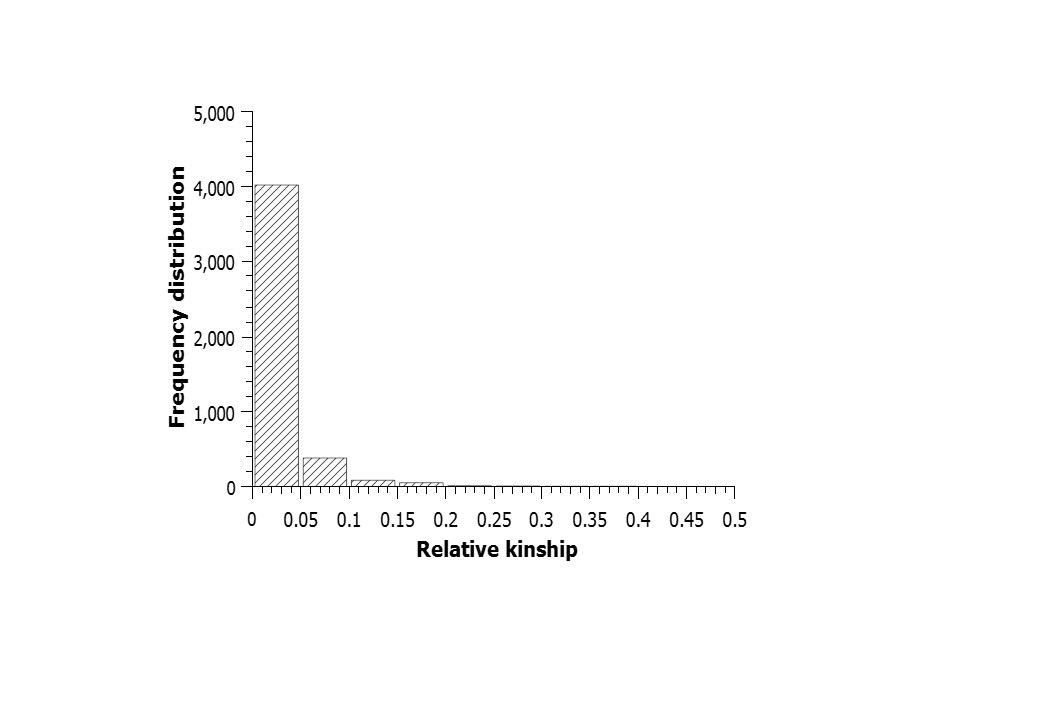

Supplement: Figure S4 — Distribution of relatedness in the association panel estimated in SPAGeDi. [file Image4.JPEG]

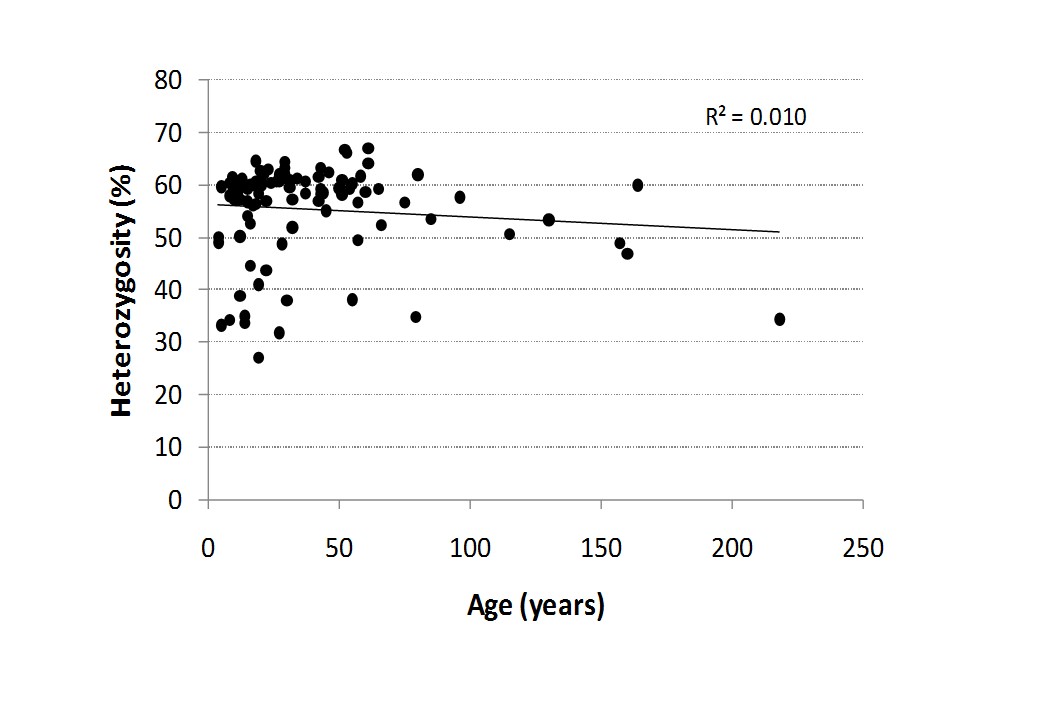

Supplement: Figure S5 — Heterozygosity of the 96 rose varieties of the association panel plotted against the age of the varieties. [file Image5.JPEG]

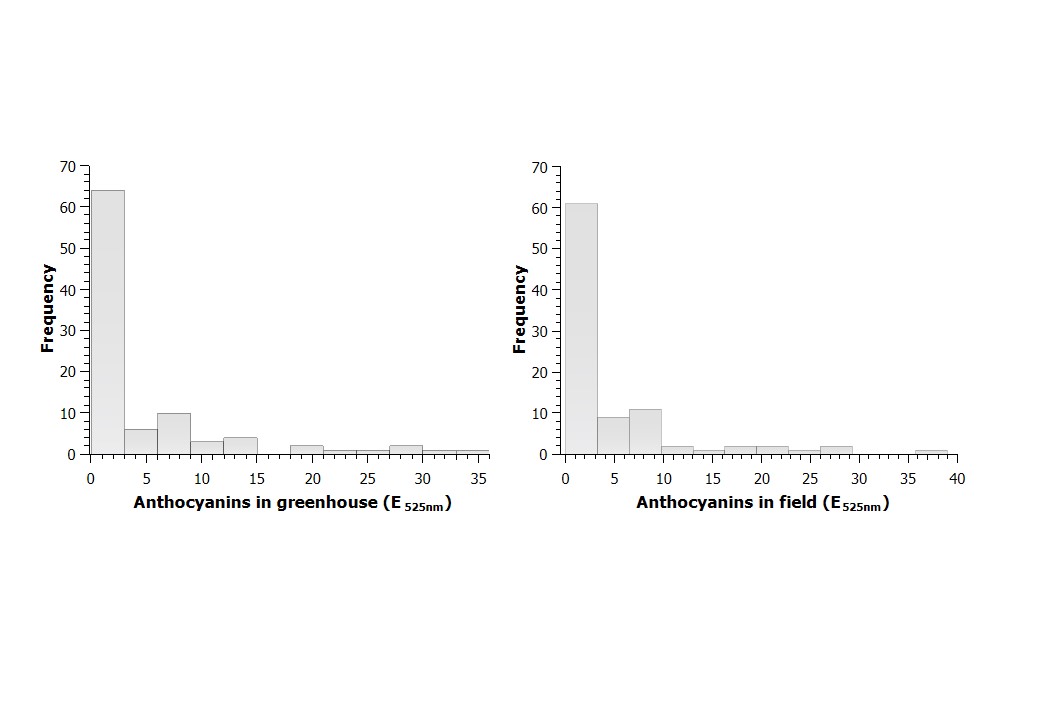

Supplement: Figure S6 — Frequency distribution of the total amount of anthocyanins in the greenhouse at the Federal Plant Variety Office (left) and in the field at Herrenhausen (right). [file Image6.JPEG]

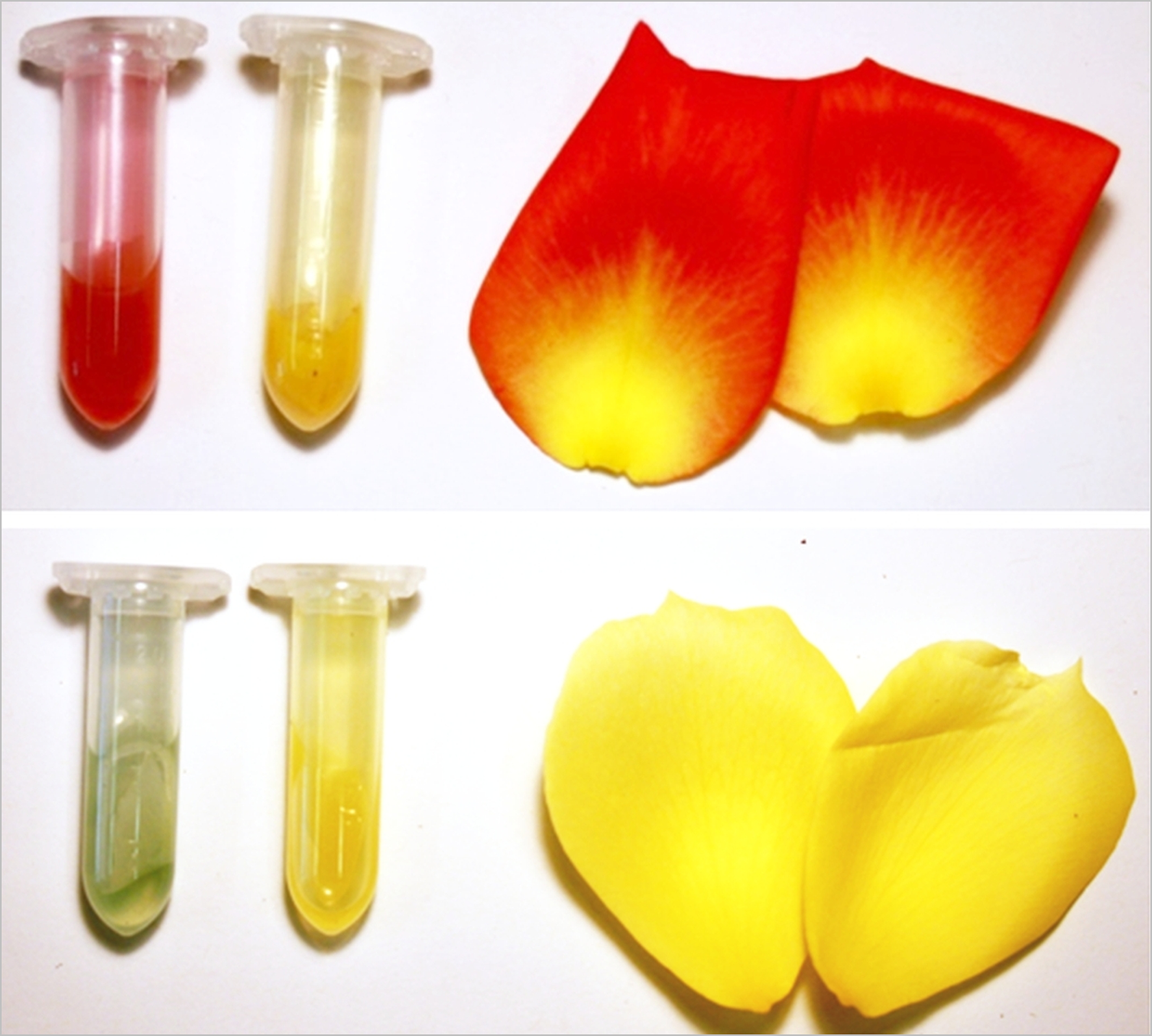

Supplement: Figure S7 — Extracts from rose petals containing anthocyanins and carotenoids (above) and rose leaves containing carotenoids only (below). [file Image7.TIF]

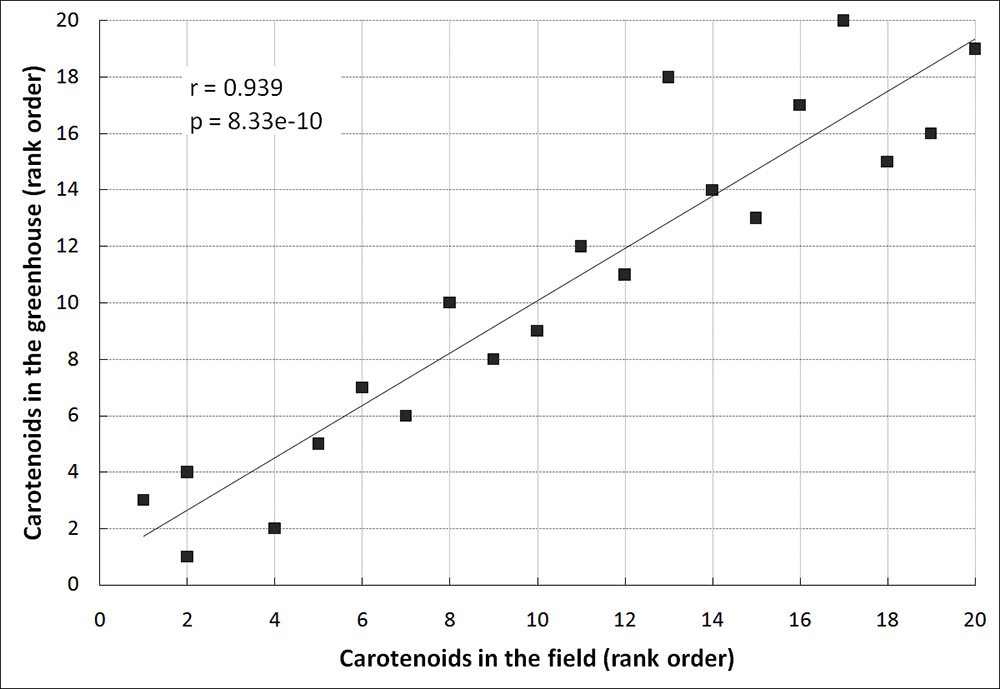

Supplement: Figure S8 — Spearman rank correlation between the total amount of carotenoids in rose petals from 20 cultivars grown in the field and in the greenhouse (Spearman's rho = 0.939). [file Image8.TIF]
