# Supplementary material for: Genome-Wide Association Analysis of the Anthocyanin and Carotenoid Contents of Rose Petals
Source: Front Plant Sci. 2016 Dec 6;7:1798. doi: 10.3389/fpls.2016.01798 (PMC5138216; doi:10.3389/fpls.2016.01798)
Supplement: Table S1 — Rose Association panel: Cultivars, breeder, origin, and breeding year of roses, code number (1–141) and flower color. Cultivars with ploidy levels differing from tetraploid are labeled as (1) or (2). [file Table1.DOCX]

**Table S1.** Rose Association panel: Cultivars, breeder, origin and breeding year of roses, code number(1 to 141) and flower colour. Cultivars with ploidy levels differing from tetraploid are labelled as ^1)^ or ^2)^.

| **Code** | **Cultivar** | **Breeder** | **Country** | **Bred in (Y)** | **Type/habit** | **Flower** |
| --- | --- | --- | --- | --- | --- | --- |
| 1 | Parole | W. Kordes&Söhne | GER | 1991 | Hybrid Tea | pink |
| 2 | Queen Elizabeth | Lammerts | USA | 1954 | Grandiflora, shrub | pink |
| 3 | Schneewittchen^1)^ | W. Kordes&Söhne | GER | 1958 | Floribunda, shrub | white |
| 4 | Nemo | Noack Rosen | GER | 2001 | Floribunda, ground cover | white |
| 5 | Super Star^1)^ | Rosen Tantau | GER | 1960 | Hybrid Tea | salmon pink |
| 6 | Small Maid. Blush | Unknown | UK | 1797 | Alba, shrub | light pink |
| 10 | Chippendale | Rosen Tantau | GER | 2005 | Hybrid Tea | orange |
| 11 | Climbing Allgold | Douglas L. Gandy | UK | 1961 | Floribunda, climber | yellow |
| 12 | Blue Parfum | Rosen Tantau | GER | 1978 | bedding | violet |
| 13 | Feuerwerk | Rosen Tantau | GER | 1962 | shrub | orange, red |
| 14 | Gebrüder Grimm | W. Kordes&Söhne | GER | 2007 | Floribunda, bedding | orange |
| 15 | George Vancouver | Ag Can | CAN | 1983 | Hybrid Kordesii, shrub | red |
| 16 | König Stanislaus | Rosen Tantau | GER | 1998 | shrub | yellow |
| 17 | Heidi Klum | Rosen Tantau | GER | 1999 | Floribunda, bedding | violet |
| 18 | Jasmina | W. Kordes&Söhne | GER | 1996 | climber | pink |
| 20 | Sonnenschirm | Rosen Tantau | GER | 1993 | Floribunda, ground cover | yellow |
| 24 | Heidetraum^1)^ | Noack Rosen | GER | 1988 | ground cover | carmine-pink |
| 26 | Nostalgie | Rosen Tantau | GER | 1995 | Hybrid Tea | white, pink |
| 27 | Sommerwind^1)^ | W. Kordes&Söhne | GER | 1985 | bedding | light pink |
| 28 | New Dawn^1)^ | Somerset Rose Nurs. | USA | 1930 | climber | light pink |
| 32 | Mevrouw N. Nypels^2)^ | Mathias Leenders | NL | 1919 | Polyantha, shrub | pink |
| 35 | Mitsouko | Delbard | F | 1970 | Hybrid Tea | yellow |
| 36 | Black Baccara | Meilland | F | 2000 | Hybrid Tea | red |
| 37 | Alinka | Patrick Dickson | UK | 1971 | Hybrid Tea | red |
| 38 | Auslo (=Othello) | David Austin Roses | UK | 1986 | shrub | red |
| 39 | Ausmas (=Graham Thomas) | David Austin Roses | UK | 1983 | shrub | yellow |
| 40 | Shalom | PoulsenRoser A/S | DAN | 1972 | Floribunda, shrub | red |
| 41 | La Sevillana | Meilland | F | 1978 | Floribunda, shrub | red |
| 42 | Mister Lincoln | Swim & Weeks | USA | 1964 | Hybrid Tea | red |
| 43 | Rumba | PoulsenRoser A/S | DAN | 1958 | Floribunda, bedding | orange |
| 44 | Arthur Bell | Sam McGredy Roses | NZ | 1965 | Floribunda, shrub | yellow |
| 46 | Comtesse de Ségur | Delbard | F | 1992 | Floribunda, shrub | pink |
| 47 | Mme Boll | Daniel Boll | USA | 1858 | Portland, shrub | red |
| 49 | Compassion | Harkness & Co Ltd. | UK | 1972 | climber | salmon-pink |
| 50 | Sutters Gold | Herbert C. Swim | USA | 1950 | Hybrid Tea | yellow |
| 51 | Scarlet Meidilland | Meilland | F | 1987 | shrub, ground cover | red |
| 52 | Rose de Resht |  | Persia | 1900 | Damask, shrub | red |
| 53 | Celine Delbard | Delbard | F | 1986 | Floribunda, shrub | salmon-pink |
| 54 | Louise Odier | Jules Margottin Père & Fils | F | 1851 | Bourbon, shrub | deep pink |
| 55 | Ausfather (=Charles Austin) | David Austin Roses | UK | 1973 | shrub | apricot |
| 56 | Perpetually Yours | Harkness & Co Ltd. | UK | 1999 | climber | light yellow |
| 57 | Mme Knorr | Viktor Verdier | F | 1855 | Portland, shrub | pink |
| 58 | Papageno | Sam McGredy Roses | NZL | 1989 | Hybrid Tea | red bled, stripes |
| 59 | France Libre | Delbard | F | 1981 | Hybrid Tea | orange |
| 61 | Princess Alexandra | PoulsenRoser A/S | DK | 1988 | Hybrid Tea | violet |
| 62 | Mrs John Laing | Henry Bennet | UK | 1885 | Hybrid Perpetual, shrub | deep pink |
| 66 | Black Magic | Rosen Tantau | GER | 1995 | Hybrid Tea | dark red |
| 67 | China Girl | Mehring/ Tantau | GER | 2005 | Floribunda, bedding | yellow |
| 68 | Perennial Blush | Henry Bennet | UK | 2007 | climber/rambler | white, light pink |
| 69 | Comtessa AL | Rosen Tantau | GER | 2006 | Hybrid Tea | yellow, white |
| 70 | Lipstick | Rosen Tantau | GER | 2001 | ground cover | pink |
| 71 | Midsummer | Rosen Tantau | GER | 2007 | Floribunda, bedding | orange-red |
| 72 | Arabia | Rosen Tantau | GER | 2001 | shrub | orange blend |
| 73 | Hansestd. Rostock | Rosen Tantau | GER | 2004 | Floribunda, bedding | apricot |
| 74 | Kastelrut. Spatzen | Rosen Tantau | GER | 2011 | ground cover | white |
| 75 | Elfe | Rosen Tantau | GER | 2000 | climber | yellow |
| 77 | Jazz | Rosen Tantau | GER | 2003 | ground cover | copper-orange |
| 78 | MainzerFastnacht | Rosen Tantau | GER | 1964 | Hybrid Tea | violet |
| 79 | Dukat | Rosen Tantau | GER | 2010 | Floribunda, climber | yellow |
| 80 | My Girl | Rosen Tantau | GER | 2006 | Hybrid Tea | white, yellow center |
| 81 | Mariatheresia | Rosen Tantau | GER | 2003 | Floribunda, bedding | light pink |
| 84 | Knockout^1)^ | Radler | USA | 1988 | shrub | red |
| 85 | Berolina | W. Kordes&Söhne | GER | 1984 | Hybrid Tea | yellow |
| 89 | Westerland | W. Kordes&Söhne | GER | 1969 | shrub | orange |
| 92 | Frühlingsduft | W. Kordes&Söhne | GER | 1949 | shrub | white, pink shading |
| 93 | Sebastian Kneipp | W. Kordes&Söhne | GER | 1997 | Hybrid Tea | white, pink center |
| 94 | Lavender Lassie^1)^ | W. Kordes&Söhne | GER | 1960 | shrub | violet |
| 95 | Dortmund | W. Kordes&Söhne | GER | 1955 | climber | red |
| 96 | Friesia | W. Kordes&Söhne | GER | 1973 | Floribunda, bedding | yellow |
| 97 | Sterntaler | W. Kordes&Söhne | GER | 1995 | shrub | yellow |
| 99 | Raubritter^1)^ | W. Kordes&Söhne | GER | 1936 | climber | light pink |
| 100 | Herkules | W. Kordes&Söhne | GER | 2006 | shrub | pink, light lavender |
| 103 | Fritz Nobis | W. Kordes&Söhne | GER | 1940 | shrub | rose-pink |
| 104 | Beverly | W. Kordes&Söhne | GER | 1999 | Hybrid Tea | pink |
| 105 | Juanita | W. Kordes&Söhne | GER | 1996 | mini-shrub | light pink |
| 110 | Windrose | Noack Rosen | GER | 1993 | ground cover | pink |
| 111 | Donauprinzessin | Noack Rosen | GER | 1994 | Floribunda, bedding | salmon-pink |
| 112 | Münsterland | Noack Rosen | GER | 1986 | Floribunda, shrub | light pink |
| 114 | Venice | Noack Rosen | GER | 2003 | Floribunda, ground cover | white |
| 115 | Focus | Noack Rosen | GER | 1997 | Hybrid Tea | light pink |
| 116 | Simply | Noack Rosen | GER | 2003 | ground cover | pink |
| 118 | Kronjuwel | Noack Rosen | GER | 1997 | Floribunda, bedding | red |
| 119 | Tornella | Noack Rosen | GER | 2005 | shrub | red |
| 120 | Herzogin Friederike | Noack Rosen | GER | 2002 | shrub | pink |
| 122 | Blue River | W. Kordes&Söhne | GER | 1984 | Hybrid Tea | magenta |
| 131 | Cute Haze | Rosen Tantau | GER | 2010 | ground cover, shrub | white |
| 132 | Duftwolke | Rosen Tantau | GER | 1963 | bedding | red |
| 133 | Goethe Rose | Rosen Tantau | GER | 2004 | Hybrid Tea | red |
| 134 | Albrecht Dürer Rose | Rosen Tantau | GER | 1996 | Hybrid Tea | orange |
| 135 | Stadt Rom | Rosen Tantau | GER | 2000 | ground cover | carmine-pink |
| 136 | Bienenweide | Rosen Tantau | UK | 2011 | mini-shrub | red |
| 137 | Lolita | W. Kordes&Söhne | GER | 1972 | Hybrid Tea | apricot |
| 138 | Magenta | W. Kordes&Söhne | GER | 1954 | Floribunda, shrub | violet |
| 139 | Rose Gaujard | Jean-Marie Gaujard | F | 1957 | Hybrid Tea | cherry-red |
| 140 | Crimson Glory | W. Kordes&Söhne | GER | 1935 | Hybrid Tea | purple, crimson |
| 141 | Sunset Boulevard | Harkness & Co Ltd. | UK | 1997 | Floribunda, shrub | salmon-pink |
