# Supplementary material for: Genome-Wide Association Analysis of the Anthocyanin and Carotenoid Contents of Rose Petals
Source: Front Plant Sci. 2016 Dec 6;7:1798. doi: 10.3389/fpls.2016.01798 (PMC5138216; doi:10.3389/fpls.2016.01798)
Supplement: Table S2 — Primer sequences, annealing temperature and linkage map location in rose (according to Spiller et al. 2011) of 27 SSR markers used in the association studies with the 96 rose cultivars. Rh primers are from Esselink et al. (2003); RMS primers are published at http://www.wipo.int/pctdb/en/wo.jsp?wo=2003097869&IA=WO2003097869&DISPLAY=STATUS. [file Table2.DOCX]

**Table S2**. Primer sequences, annealing temperature and linkage map location in rose (according to Spiller et al. 2011) of 27 SSR markers used in the association studies with the 96 rose cultivars. Rh primers are from Esselink et al. (2003); RMS primers are published at http://www.wipo.int/pctdb/en/wo.jsp?wo=2003097869&IA=WO2003097869&DISPLAY=STATUS.

| **SSR primer** | **Sequence of forward primer  (5'--3')** | **Sequence of reverse primer  (5'--3')** | **Annealing T. (°C)** | **Linkage group** |
| --- | --- | --- | --- | --- |
| RhAB38 | GAGGTGGTCGATTCCATGTC | TTACCGTTCTACCTAAGTGACTAAC | 50 | 5 |
| Rh50 | TGATGAAATCATCCGAGTGTCAG | TCACTTTCATTGGAATGCCAGAAT | 50 | 3 |
| Rh58 | ACAATTTAGTGCGGATAGAACAAC | GGAAAGCCCGAAAGCGTAAGC | 50 | 3 |
| Rh73 | GGTTAGACGGGTGGAAGAAG | ACTGCCGATAGAAGTATTTCATCA | 50 | 7 |
| Rh79 | TTCTTCTTGCTCGCCATTTTGATT | GAACGTCCACCACCACCCACTCTG | 50 | 1 |
| Rh80 | CATGCCAAACGAAATGAGTTA | TTATCTAAAGGGCTGCTGTAAGTT | 50 | 2 |
| RMS011 | TAGAAACGACCAATAAAAGAGG | TAACGAAACATCATCAATAGCA | 55 | nd |
| RMS015 | TAATGTAGGCAGATATAAAGGAGT | GCAGCTGCACAACAAGGAA | 55 | 1 |
| RMS024 | ACTACTGTAAAATATGAAAAATCC | GTAGTAGCGGTTGCAAGAAAATA | 55 | nd |
| RMS030 | GATAAATTTCAAGGCGAGAG | AAAAGATGAACGACCCAAATAAT | 55 | nd |
| RMS038 | GTGATAAGAGCAAAACAAGATGG | CTCGCGGAAGCCTCAAAA | 55 | nd |
| RMS043 | GATCAAAGATGGGTTCTCCTCTC | AGGGGAATCTTTGAAAGTCGTTC | 60 | 7 |
| RMS045 | GAAAATAAGGACATCATCTAC | GGTGCCTCCATTATTTAC | 55 | 5 |
| RMS047 | GCTCCCTCAATTTCCACTCA | ACCAACCCAATTCGCTCAT | 60 | nd |
| RMS055 | TGATCACAAGAGCTTTTCAAGTTTAG | AGTTAGGCGCATGTACAAGAAAAT | 55 | nd |
| RMS058 | CAACCCCTGAAGCCTGAA | TTTGTAACCCATTTGACCATA | 55 | nd |
| RMS060 | CATTCATTTGACTCTAAGGA | TATTCTGGTCTAAGCTATTGTAA | 50 | 1 |
| RMS065 | TATAGCTCGGTAGATTCAAA | CCAGACTGCCCCCAACTCATA | 55 | 2 |
| RMS066 | TCCACCCACAGACCACAG | AAGCTCCCTACGATTTCACTC | 60 | 7 |
| RMS070 | TGCCTCTCGATACAAACC | AATAAGAACCAATACCCCGAAGAG | 50 | 1 |
| RMS071 | GTTAGCATCTGGCACATTAT | AGTTCCTTGACCAGCAGAG | 55 | nd |
| RMS072 | TTAGCTCAAGAATTCATCAAAG | TCCAAACCGAGCTAAGAAAACT | 55 | nd |
| RMS073 | AAACCCCTTTTATGTAGAAGTAG | TAAAACATGAAATTATAACAATAGTG | 50 | nd |
| RMS077 | AGGTGAACATGGGCCAACTA | TCAAAGAATGAGTGCCTACTAAGA | 60 | nd |
| RMS079 | CCGGTATGGAGAGGAATGAG | GCAATTATCCTTGACAGAACCC | 60 | nd |
| RMS082 | AACAACACACGCGGAATATG | TGCAGTTGGAGTTGGAGTTG | 55 | nd |
| RMS091 | GATCAGGGTGAAtACCGAGC | GCCACTCTTCTCTGTCCTCAA | 55 | 4 |
