# Supplementary material for: Genome-Wide Association Analysis of the Anthocyanin and Carotenoid Contents of Rose Petals
Source: Front Plant Sci. 2016 Dec 6;7:1798. doi: 10.3389/fpls.2016.01798 (PMC5138216; doi:10.3389/fpls.2016.01798)
Supplement: Table S3 — Candidate genes for anthocyanin biosynthesis localized in the genomes of F. vesca and P. persica. [file Table3.DOCX]

**Table S3.** Candidate genes for anthocyanin biosynthesis localized in the genomes of *F. vesca* and *P. persica*

| **Anthocyanin** | ***Fragaria vesca*** | | ***Prunus persica*** | |  | **SNP** | **Function** |
| --- | --- | --- | --- | --- | --- | --- | --- |
| Candidate gene | LG | Position | LG | Position | p-value |  |  |
| GST | Fvb1 | 13457300 | Pp03 | 958147 | 1.23E-07 | RhK5_7371_202Q | gene31672-v1.0-hybrid_Glutathione_S-transferase_(similar_to) |
| E3_C8 | Fvb1 | 13457759 | -- | -- | 4.08E-05 | RhK5_11612_458Q | gene08542-v1.0-hybrid_E3_ubiquitin-protein_ligase_CIP8_(similar_to) |
| SUMO1 | Fvb1 | 13495336 | -- | -- | 8.45E-07 | Rh12GR_17814_425Q | gene31679-v1.0-hybrid_Ubiquitin-like_protein_SMT3_(probable) |
| ARF_8 | Fvb1 | 13602666 | Pp03 | 731901 | 9.73E-11 | Rh12GR_283_1910Q | gene31631-v1.0-hybrid_Auxin_response_factor_8_(putative) |
|  | Fvb3 | 2887619 | Pp04 | 4220574 | 9.73E-11 |  |  |
| ABC_E | Fvb2 | 14129229 | Pp03 | 1321116 | 1.73E-05 | Rh12GR_25909_1582P | gene08651-v1.0-hybrid_ABC_transporter_E_family_member_2  _ (ABC_transporter_ABCE.2)_(putative) |
| IAA_S | Fvb2 | 20190055 | Pp08 | 21927020 | 2.20E-06 | Rh12GR_3292_1365P | gene10566-v1.0-hybrid_Putative_indole-3-acetic_acid-amido_ synthetase_GH3.9_(AtGH3-9) |
| Cirhin/WD40 | Fvb2 | 24397420 | Pp01 | 36401229 | 8.10E-09 | RhK5_1439_806P | gene02598-v1.0-hybrid_Cirhin_(probable) U3 small nucleolar RNA-associated protein 4/UTP4 |
| WRKY17 | Fvb2 | 26458029 | Pp01 | 38446355 | 8.74E-06 | RhK5_9709_542P | gene08720-v1.0-hybrid_Probable_WRKY_transcription_factor_17_ (putative) |
| ABC_G | Fvb2 | 26806823 | Pp01 | 32869706 | 3.50E-05 | RhK5_3307_1142Q | gene08654-v1.0-hybrid_ABC_transporter_G_family_member_14_ (ABC_transporter_ABCG.14)_(similar_to) |
| ABC_E | Fvb3 | 1748904 | Pp01 | 33582218 | 1.73E-05 | Rh12GR_25909_1582P | gene08651-v1.0-hybrid_ABC_transporter_E_family_member_2_ (ABC_transporter_ABCE.2)_(putative) |
| E3_RNF8 | Fvb4 | 24461428 | Pp01 | 25316517 | 2.39E-05 | RhK5_20085_328Q | gene06939-v1.0-hybrid_E3_ubiquitin-protein_ligase_RNF8_A_ (probable) |
| 4_CL | Fvb4 | 24864120 | Pp01 | 24722871 | 6.58E-06 | RhK5_17800_191Q | gene07129-v1.0-hybrid_4-coumarate--CoA_ligaselike_9_ (At4CL4)_(probable) |
| E3_UBR7 | Fvb5 | 2655879 | Pp05 | 12015565 | 4.44E-05 | RhK5_6420_651Q | gene32102-v1.0-hybrid_Putative_E3_ubiquitin-protein_ligase_UBR7_ (probable) |
| F3'H | Fvb5 | 8096310 | Pp05 | 16349509 | 3.44E-05 | RhK5_2457_1244P | gene25801-v1.0-hybrid_Flavonoid_3'-monooxygenase_(similar_to) |
| GT83a | Fvb5 | 8166928 | Pp05 | 12015565 | 4.44E-05 | RhK5_19460_153 | gene04292-v1.0-hybrid_Cytokinin-O-glucosyltransferase_2_ (AtZOG2)_ (probable) - udp-glycosyltransferase 83a1-like |
| Myb90 | Fvb6 | 630966 | Pp06 | 30200282 | 3.55E-06 | RhMCRND_10092_296P | gene25801-v1.0-hybrid_Flavonoid_3'-monooxygenase_(similar_to) |
| E3_C8 | Fvb6 | 30195732 | -- | -- | 3.42E-05 | Rh12GR_22762_321P | gene04302-v1.0-hybrid_E3_ubiquitin-protein_ligase_CIP8_(probable) |
