# Supplementary material for: Genome-Wide Association Analysis of the Anthocyanin and Carotenoid Contents of Rose Petals
Source: Front Plant Sci. 2016 Dec 6;7:1798. doi: 10.3389/fpls.2016.01798 (PMC5138216; doi:10.3389/fpls.2016.01798)
Supplement: Table S4 — Candidate genes for carotenoid biosysnthesis in the genomes of F. vesca and P. persica. [file Table4.DOCX]

**Table S4.** Candidate genes for carotenoid biosysnthesis in the genomes of *F. vesca* and *P. persica*

| **Carotenoid** | ***Fragaria vesca*** | | ***Prunus persica*** | | | **SNP** | **Function** |
| --- | --- | --- | --- | --- | --- | --- | --- |
| **Candidate gene** | LG | Position | LG | Position | p-value |  |  |
| **Pmzeaep** | Fvb1  Fvb6 | 9801058  37472751 | Pp07 | 15160943 | 8.77e-06 | RhK5_3146_112P | Zeaxanthin_epoxidase,_chloroplastic,_Precursor_(similar) |
| **CYP_749A22** | Fvb4 | 28195854 | Pp01  Pp05  Pp08 | 29269183  5005481  1978005 | 9.34E-07 | Rh12GR_26776_1835P | Cytochrome P450 cyp749a22-like |
| **UBL_E3** | Fvb5 | 1638614 | Pp05 | 11049604 | 2.53E-09 | Rh12GR_5175_1076P | e3 ubiquitin-protein ligase at3g02290 |
| **Myb86** | Fvb5 | 1824383 | Pp05 | 11209101 | 4.12E-10 | Rh12GR_17126_1100P | transcription factor myb86 |
| **CMS** | Fvb5 | 1850716 | Pp05 | 11248627 | 1.11E-10 | RhK5_6118_534P | 2-c-methyl-d-erythritol -cyclodiphosphate chloroplastic |
| **SUMO_PLI1** | Fvb5 | 1883476 | Pp05 | 12174164 | 1.39E-09 | RhK5_9077_390Q | e3 sumo-protein ligase pli1 |
| **E3_UBR7** | Fvb5 | 2655879 | Pp05 | 12015565 | 2.25E-09 | Rh12GR_25827_1930P | e3 ubiquitin-protein ligase ubr7 |
| **E3_M10** | Fvb5 | 2876834 | Pp05 | 11290885 | 2.90E-08 | RhK5_7624_963P | e3 ubiquitin-protein ligase march10 |
| **UBC_E2** | Fvb5 | 2908194 | Pp05 | 14981826 | 1.31E-07 | RhK5_6336_579P | ubiquitin-conjugating enzyme e2-23 kda-like |
| **DXR** | Fvb5  Fvb3 | 3041713  20970223 | Pp05 | 14834185 | 2.52E-08 | RhK5_2653_1303Q | 1-deoxy-d-xylulose 5-phosphate reductoisomerase |
| **CYP_71A25** | Fvb5 | 4300707 | Pp04 | 15705728 | 1.16e-10 | RhMCRND_4851_1084Q | Cytochrome_ P450_71A25_(similar_to) |
| **TF26** | Fvb5 | 13095845 | Pp05 | 6296596 | 2.08E-08 | RhK5_2968_844P | gata transcription factor 26-like |
