# Supplementary material for: Genome-Wide Association Analysis of the Anthocyanin and Carotenoid Contents of Rose Petals
Source: Front Plant Sci. 2016 Dec 6;7:1798. doi: 10.3389/fpls.2016.01798 (PMC5138216; doi:10.3389/fpls.2016.01798)
Supplement: Table S5 — SNPs significantly associated with the anthocyanin and carotenoid contents after Bonferroni correction at a significance level of α = 2.62E-6. [file Table5.docx]

**Table S5.** SNPs significantly associated with carotenoid content after Bonferroni correction at a significance level of α = 2.62E-6.

| **Trait** | **SNP** | **p-value** | **Effect^1)^(E_442nm_)** | **Allele A:A** | **Allele A:B** | **Allele B:B** |
| --- | --- | --- | --- | --- | --- | --- |
| Carotenoids | Rh12GR_3624_1035Q | 2.10E-10 | 0.102 | - | 54 | 29 |
| Carotenoids | RhK5_1045_1129Q | 2.43E-12 | -0.129 | - | 44 | 47 |
| Carotenoids | RhK5_1614_981P | 1.20E-08 | -0.127 | - | 28 | 63 |
| Carotenoids | RhK5_222_1140Q | 9.37E-09 | -0.097 | 51 | 42 | - |
| Carotenoids | RhK5_718_563P | 4.24E-11 | 0.119 | - | 47 | 45 |
| Carotenoids | RhK5_7528_1015P | 2.68E-09 | -0.178 | - | 23 | 61 |
| Carotenoids | RhK5_7755_2216Q | 5.77E-09 | 0.098 | - | 47 | 44 |
| Carotenoids | RhMCRND_4851_1084Q | 1.16E-10 | -0.003 | - | 44 | 48 |

^1)^untransformed values.
