# Supplementary material for: Genome-Wide Association Analysis of the Anthocyanin and Carotenoid Contents of Rose Petals
Source: Front Plant Sci. 2016 Dec 6;7:1798. doi: 10.3389/fpls.2016.01798 (PMC5138216; doi:10.3389/fpls.2016.01798)
Supplement: Table S6 — Anthocyanin SNP markers and their position on the genome of Fragaria vesca 2.0. [file Table6.DOCX]

**Table S6.** Anthocyanin SNP markers and their position on the genome of *Fragaria vesca* 2.0.

| SNP | LG (*F. vesca*) | Position | p-value | Gene (Function) |
| --- | --- | --- | --- | --- |
| Rh12GR_92431_4144Q | Fvb1 | 13267031 | 8.48E-07 | Serine/threonine-protein_kinase_PBS1_ |
| RhMCRND_7128_1021Q | Fvb1 | 13435993 | 9.15E-06 | Medium-chain-fatty-acid--CoA_ligase_ |
| Rh12GR_20064_1031P | Fvb1 | 13442276 | 1.21E-07 | Medium-chain-fatty-acid--CoA_ligase_ |
| RhMCRND_20203_163Q | Fvb1 | 13444165 | 1.17E-08 | Medium-chain-fatty-acid--CoA_ligase_ |
| RhK5_7371_202Q | Fvb1 | 13457300 | 1.23E-07 | Glutathione_S-transferase_(similar_to) |
| RhK5_11612_458Q | Fvb1 | 13457759 | 4.08E-05 | E3_ubiquitin-protein_ligase_CIP8_(similar_to) |
| RhMCRND_6329_536P | Fvb1 | 13458969 | 1.67E-05 | F-box/kelch-repeat_protein_At3g23880_ |
| RhMCRND_633_2817P | Fvb1 | 13458969 | 4.26E-05 | Filament-like_plant_protein_7_(AtFPP7)_ |
| Rh12GR_77973_217Q | Fvb1 | 13476535 | 4.54E-06 | -- |
| Rh12GR_17814_425Q | Fvb1 | 13495336 | 8.45E-07 | Ubiquitin-like_protein_SMT3_ |
| Rh12GR_17814_425P | Fvb1 | 13495336 | 1.19E-05 | Ubiquitin-like_protein_SMT3_ |
| RhMCRND_24509_109Q | Fvb1 | 13595734 | 3.85E-05 | Auxin_response_factor_8_ |
| Rh12GR_283_1910Q | Fvb1 | 13602666 | 9.73E-11 | Auxin_response_factor_8_ |
| Rh12GR_283_1910P | Fvb1 | 13602666 | 7.76E-06 | Auxin_response_factor_8_ |
| RhK5_15649_288P | Fvb1 | 13821855 | 7.22E-06 | -- |
| RhK5_12663_103Q | Fvb1 | 13924865 | 1.37E-06 | Possible_hemolysin_C_ |
| RhK5_12663_103P | Fvb1 | 13924865 | 1.31E-05 | Possible_hemolysin_C_ |
| RhMCRND_5766_773P | Fvb1 | 14029764 | 3.09E-05 | tRNA_guanosine-2'-O-methyltransferase_TRM13 _homolog_ |
| RhK5_143_3043Q | Fvb1 | 15025558 | 5.44E-05 | Phosphatidylinositol_4-kinase_(PtdIns-4-kinase) _(similar_to) |
| RhMCRND_7194_1031P | Fvb1 | 17018632 | 4.82E-05 | -- |
| Rh12GR_35622_191Q | Fvb1 | 17680092 | 2.50E-05 | DEAD-box_ATP-dependent_RNA_helicase_24_ |
| Rh12GR_72173_3162P | Fvb1 | 17703044 | 2.54E-05 | Nodal_modulator_1,_Precursor_ |
| RhK5_1023_664P | Fvb1 | 17717891 | 1.06E-05 | Pentatricopeptide_repeat-containing_protein_ At2g31400_chloroplastic, _Precursor_(similar_to) |
| RhK5_1023_664Q | Fvb1 | 17717891 | 2.49E-05 | Pentatricopeptide_repeat-containing_protein_ At2g31400_chloroplastic, _Precursor_(similar_to) |
| RhK5_8234_337Q | Fvb1 | 17777094 | 4.07E-05 | Flowering_time_control_protein_FCA_ |
| RhK5_661_2871P | Fvb2 | 8403283 | 4.85E-05 | Cell_division_cycle_protein_48_homolog_(VCP) |
| Rh12GR_25909_1582P | Fvb2 | 14129229 | 1.73E-05 | ABC_transporter_E_family_member_2_(ABCE.2) |
| Rh12GR_25909_1582Q | Fvb2 | 14129229 | 4.78E-05 | ABC_transporter_E_family_member_2_(ABCE.2) |
| RhMCRND_9275_1067P | Fvb2 | 17138575 | 2.78E-05 | hypothetical_protein |
| Rh12GR_3292_1365P | Fvb2 | 20190055 | 2.20E-06 | Putative_indole-3-acetic_acid-amido_synthetase_ GH3.9_(AtGH3-9) |
| RhMCRND_3273_1540Q | Fvb2 | 21900253 | 8.53E-06 | ATP_synthase_subunit_alpha,_mitochondrial_  (similar_to) |
| RhMCRND_1270_967P | Fvb2 | 23654492 | 5.21E-06 | Puromycin-sensitive_aminopeptidase_(PSA) |
| RhK5_1439_806P | Fvb2 | 24397420 | 8.10E-09 | U3 small nucleolar RNA-associated protein 4/UTP4 |
| RhK5_2615_1401Q | Fvb2 | 24936235 | 8.64E-07 | Cysteine_proteinase_RD21a_(RD21),_Precursor |
| RhK5_7441_226P | Fvb2 | 26388093 | 5.67E-06 | Probable_xyloglucan_glycosyltransferase_5_AtCslC5 |
| RhK5_7441_226Q | Fvb2 | 26388093 | 1.18E-05 | Probable_xyloglucan_glycosyltransferase_5_AtCslC5 |
| RhK5_9709_542P | Fvb2 | 26458029 | 8.74E-06 | Probable_WRKY_transcription_factor_17_ |
| RhK5_1258_2078P | Fvb2 | 26605012 | 9.44E-08 | 3beta-hydroxysteroid-dehydrogenase/decarboxylase _isoform_2_(At3BETAHSD/D2)_(similar_to) |
| RhK5_3307_1142Q | Fvb2 | 26806823 | 3.50E-05 | ABC_transporter_G_family_member_14_(ABCG.14) |
| RhK5_3307_1142P | Fvb2 | 26806823 | 4.70E-05 | ABC_transporter_G_family_member_14_(ABCG.14) |
| RhMCRND_4945_1158P | Fvb2 | 26990719 | 1.42E-05 | Lysine-specific_histone_demethylase_1_ homolog_1 |
| RhMCRND_4945_1158Q | Fvb2 | 26990719 | 3.91E-05 | Lysine-specific_histone_demethylase_1_ homolog_1 |
| Rh12GR_76625_2294P | Fvb2 | 26990719 | 5.34E-05 | Lysine-specific_histone_demethylase_1_ homolog_1 |
| RhK5_11612_458Q | Fvb2 | 27332875 | 4.08E-05 | E3_ubiquitin-protein_ligase_CIP8_(similar_to) |
| RhMCRND_8602_351Q | Fvb2 | 27586474 | 2.92E-05 | Probable_E3_ubiquitin-protein_ligase_ARI5 |
| RhMCRND_8602_351P | Fvb2 | 27586474 | 4.26E-05 | Probable_E3_ubiquitin-protein_ligase_ARI5 |
| Rh12GR_37046_341Q | Fvb2 | 27737487 | 1.01E-05 | Kinesin-related_protein_4_ |
| Rh12GR_67652_1670P | Fvb2 | 27740213 | 2.21E-05 | Kinesin-related_protein_4_ |
| Rh12GR_67652_1670Q | Fvb2 | 27740213 | 3.59E-05 | Kinesin-related_protein_4_ |
| RhK5_983_1142Q | Fvb2 | 27742990 | 2.03E-05 | Protein_MTL1,_Precursor_ |
| Rh12GR_38264_410P | Fvb2 | 28371676 | 8.55E-06 | -- |
| Rh12GR_38264_410Q | Fvb2 | 28371676 | 4.78E-05 | -- |
| Rh12GR_56471_686P | Fvb2 | 28675593 | 4.65E-05 | Chaperone_protein_clpB_2_(similar_to) |
| RhK5_5250_853Q | Fvb3 | 352908 | 4.46E-06 | Ras-related_protein_ARA-4_(similar_to) |
| Rh12GR_25909_1582P | Fvb3 | 1748904 | 1.73E-05 | ABC_transporter_E_family_member_2_(ABCE.2) |
| Rh12GR_25909_1582Q | Fvb3 | 1748904 | 4.78E-05 | ABC_transporter_E_family_member_2_(ABCE.2) |
| Rh12GR_283_1910Q | Fvb3 | 2887619 | 9.73E-11 | Auxin_response_factor_8_ |
| Rh12GR_283_1910P | Fvb3 | 2887619 | 7.76E-06 | Auxin_response_factor_8_ |
| RhK5_1258_2078P | Fvb3 | 10339744 | 9.44E-08 | 3beta-hydroxysteroid-dehydrogenase/decarboxy lase_isoform_2_(At3BETAHSD/D2)_(similar_to) |
| Rh12GR_51872_2470P | Fvb3 | 26180214 | 1.83E-05 | Pentatricopeptide_repeat-containing_protein_ At5g27110_(similar_to) |
| RhMCRND_982_2342Q | Fvb3 | 30886399 | 8.43E-07 | Formin-like_protein_20_(AtFH20)_(similar_to) |
| RhMCRND_982_2342P | Fvb3 | 30886399 | 6.75E-06 | Formin-like_protein_20_(AtFH20)_(similar_to) |
| RhK5_252_2279P | Fvb3 | 32807322 | 1.02E-05 | TATA-binding_protein-associated_factor_172 _(TAF172) |
| Rh12GR_78838_640Q | Fvb4 | 2664856 | 4.38E-05 | -- |
| RhK5_661_2871P | Fvb4 | 19989248 | 4.85E-05 | Cell_division_cycle_protein_48_homolog_(VCP)_ |
| RhK5_14520_1662P | Fvb4 | 23323967 | 2.04E-05 | Guanosine-3',5'-bis(diphosphate)_3'-pyrophospho hydrolase_((ppGpp)ase)_ |
| RhK5_14520_1662Q | Fvb4 | 23323967 | 3.07E-05 | Guanosine-3',5'-bis(diphosphate)_3'-pyrophospho hydrolase_((ppGpp)ase)_ |
| RhMCRND_3357_1354P | Fvb4 | 24102157 | 1.18E-05 | U-box_domain-containing_protein_14_ |
| Rh12GR_29280_301Q | Fvb4 | 24141944 | 6.64E-06 | Serine/threonine-protein_kinase_AFC2_ |
| RhK5_21626_409P | Fvb4 | 24181006 | 1.66E-06 | Anthranilate_phosphoribosyltransferase_ |
| RhK5_21626_309Q | Fvb4 | 24181006 | 1.27E-05 | Anthranilate_phosphoribosyltransferase_ |
| RhK5_21626_309P | Fvb4 | 24181006 | 2.30E-05 | Anthranilate_phosphoribosyltransferase_ |
| RhK5_6000_648P | Fvb4 | 24183643 | 3.45E-05 | Anthranilate_phosphoribosyltransferase_ |
| Rh12GR_70723_479P | Fvb4 | 24261526 | 1.27E-05 | Putative_1-aminocyclopropane-1-carboxylate_deaminase_(ACC_deaminase)_ |
| RhK5_1453_1633Q | Fvb4 | 24284368 | 1.38E-05 | Actin-related_protein_3_ |
| Rh12GR_33776_266Q | Fvb4 | 24286155 | 2.47E-05 | Actin-related_protein_3_ |
| RhK5_6499_205Q | Fvb4 | 24290028 | 1.64E-05 | Multiple_RNA-binding_domain-containing_protein_1_ |
| Rh12GR_78128_147P | Fvb4 | 24295598 | 1.50E-05 | -- |
| RhMCRND_24186_368Q | Fvb4 | 24316507 | 1.65E-05 | -- |
| RhK5_4436_1053P | Fvb4 | 24338496 | 1.08E-05 | Estradiol_17-beta-dehydrogenase_12_  (17-beta-HSD_12)_ |
| RhK5_20085_328Q | Fvb4 | 24461428 | 2.39E-05 | E3_ubiquitin-protein_ligase_RNF8_A_ |
| RhMCRND_5159_1074Q | Fvb4 | 24498647 | 8.53E-06 | Probable_gibberellin_receptor_GID1L2 |
| RhK5_4451_1155Q | Fvb4 | 24626095 | 4.45E-05 | F-box_protein_SKIP14_ |
| RhK5_1176_384P | Fvb4 | 24644239 | 3.61E-05 | Serine/threonine_protein_phosphatase_2A_  59_kDa_regulatory_subunit_B'_eta_isoform_  (PP2A,_B'_subunit,_eta_isoform)_(similar_to) |
| RhMCRND_12192_640P | Fvb4 | 24721001 | 4.65E-06 | Lysine_histidine_transporter_1_ |
| RhK5_5774_854P | Fvb4 | 24841219 | 1.11E-06 | Translation_initiation_factor_IF-2_ |
| RhK5_5774_1021Q | Fvb4 | 24841219 | 3.73E-05 | Translation_initiation_factor_IF-2_ |
| RhK5_5774_1021P | Fvb4 | 24841219 | 3.82E-05 | Translation_initiation_factor_IF-2_ |
| RhK5_17800_191Q | Fvb4 | 24864120 | 6.58E-06 | 4-coumarate--CoA_ligase-like_9_(At4CL4)_ |
| RhMCRND_319_1197P | Fvb4 | 24867564 | 7.49E-07 | Urease_(similar_to) |
| RhK5_6420_651Q | Fvb5 | 2655879 | 4.44E-05 | Putative_E3_ubiquitin-protein_ligase_UBR7_ |
| RhMCRND_1270_967P | Fvb5 | 4135329 | 5.21E-06 | Puromycin-sensitive_aminopeptidase_(PSA)_ |
| RhMCRND_3654_149Q | Fvb5 | 7962396 | 4.77E-05 | Presenilin-like_protein_At2g29900_ |
| RhK5_12769_196P | Fvb5 | 7965879 | 2.21E-05 | Glycine-rich_RNA-inding_protein_2,_mitochondrial_ (AtGRP2),_Precursor_(similar_to)) |
| RhK5_12769_196Q | Fvb5 | 7965879 | 3.79E-05 | Glycine-rich_RNA-inding_protein_2,_mitochondrial_ (AtGRP2),_Precursor_(similar_to)) |
| RhK5_4231_969P | Fvb5 | 7985441 | 6.24E-06 | ATP_synthase_subunit_gamma,_mitochondrial, _Precursor_(similar_to) |
| RhK5_4231_969Q | Fvb5 | 7985441 | 1.31E-05 | ATP_synthase_subunit_gamma,_mitochondrial, _Precursor_(similar_to) |
| RhK5_2604_983Q | Fvb5 | 8032071 | 3.51E-05 | Histone_deacetylase_5_(similar_to) |
| RhK5_2604_983P | Fvb5 | 8032071 | 5.20E-05 | Histone_deacetylase_5_(similar_to) |
| RhK5_30_672Q | Fvb5 | 8072385 | 1.31E-05 | Activating_signal_cointegrator_1_complex_subunit_3_(similar_to) |
| RhK5_30_672P | Fvb5 | 8072385 | 2.21E-05 | Activating_signal_cointegrator_1_complex_subunit_3_(similar_to) |
| RhK5_2457_1244P | Fvb5 | 8096310 | 3.44E-05 | Flavonoid_3'-monooxygenase_(similar_to) |
| RhK5_2457_1244Q | Fvb5 | 8096310 | 4.25E-05 | Flavonoid_3'-monooxygenase_(similar_to) |
| RhK5_617_2964Q | Fvb5 | 8104771 | 3.47E-05 | Heat_shock_protein_101_ |
| RhK5_32_1134P | Fvb5 | 8122749 | 1.31E-05 | Dentin_sialoprotein,_Precursor_ |
| RhK5_32_1134Q | Fvb5 | 8122749 | 1.31E-05 | Dentin_sialoprotein,_Precursor_ |
| RhMCRND_37_4783Q | Fvb5 | 8122749 | 1.33E-05 | Dentin_sialoprotein,_Precursor_ |
| RhK5_32_3679P | Fvb5 | 8122749 | 1.37E-05 | Dentin_sialoprotein,_Precursor_ |
| RhMCRND_37_1580Q | Fvb5 | 8122749 | 2.88E-05 | Dentin_sialoprotein,_Precursor_ |
| RhMCRND_37_1580P | Fvb5 | 8122749 | 3.11E-05 | Dentin_sialoprotein,_Precursor_ |
| RhK5_19460_153P | Fvb5 | 8166928 | 6.85E-06 | Cytokinin-O-glucosyltransferase_2_(AtZOG2) |
| Rh12GR_24629_1591P | Fvb5 | 8214274 | 5.53E-06 | Zinc_finger_protein_1_ |
| RhMCRND_21965_337P | Fvb5 | 8249119 | 1.25E-05 | Rhamnogalacturonate_lyase_(Rhamnogalactu-  ronase),_Precursor_ |
| Rh12GR_83294_495P | Fvb5 | 8258382 | 9.14E-06 | -- |
| Rh12GR_83294_495Q | Fvb5 | 8258382 | 2.41E-05 | -- |
| Rh12GR_18342_161P | Fvb5 | 8329658 | 4.58E-06 | Intracellular_protein_transport_protein_USO1 _(Int-1) |
| Rh12GR_18342_161Q | Fvb5 | 8329658 | 1.55E-05 | Intracellular_protein_transport_protein_USO1 _(Int-1) |
| RhMCRND_1369_1182Q | Fvb5 | 8357157 | 4.94E-06 | Fragaria vesca subsp. vesca probable serine/ threonine-protein kinase At1g54610 |
| RhMCRND_982_2342Q | Fvb5 | 8363545 | 8.43E-07 | Formin-like_protein_20_(AtFH20)_(similar_to) |
| Rh12GR_18364_3187Q | Fvb5 | 8363545 | 2.83E-06 | Formin-like_protein_20_(AtFH20)_(similar_to) |
| RhMCRND_982_2342P | Fvb5 | 8363545 | 6.75E-06 | Formin-like_protein_20_(AtFH20)_(similar_to) |
| RhK5_15799_993P | Fvb5 | 8364250 | 1.00E-06 | Formin-like_protein_20_(AtFH20)_(similar_to) |
| RhK5_1553_678P | Fvb5 | 8371917 | 5.30E-06 | Formin-like_protein_20_(AtFH20)_(similar_to) |
| RhK5_1553_678Q | Fvb5 | 8371917 | 8.75E-06 | Formin-like_protein_20_(AtFH20)_(similar_to) |
| RhK5_8039_1263Q | Fvb5 | 8387148 | 2.24E-05 | Probable_receptor-like_protein_kinase_ At5g61350,_Precursor_(similar_to) |
| RhMCRND_1005_1308P | Fvb5 | 8388479 | 4.88E-05 | Probable_receptor-like_protein_kinase_ At5g61350,_Precursor_(similar_to) |
| RhK5_8047_481P | Fvb5 | 8427873 | 3.84E-05 | Two-component_response_regulator-like_APRR1 |
| Rh12GR_53541_133P | Fvb6 | 51631 | 1.46E-05 | -- |
| Rh12GR_82915_1105P | Fvb6 | 57746 | 1.64E-05 | Endoribonuclease_Dicer_homolog_2a_(OsDCL2a) |
| Rh12GR_82915_1105Q | Fvb6 | 57746 | 3.26E-05 | Endoribonuclease_Dicer_homolog_2a_(OsDCL2a) |
| RhK5_2980_574Q | Fvb6 | 74368 | 4.12E-05 | 60S_ribosomal_protein_L38_(similar_to) |
| RhK5_2980_574P | Fvb6 | 74368 | 4.15E-05 | 60S_ribosomal_protein_L38_(similar_to) |
| RhK5_456_2666Q | Fvb6 | 173382 | 1.47E-05 | Type_II_inositol-1,4,5-trisphosphate_5-phosphatase _FRA3_ |
| RhMCRND_7884_156Q | Fvb6 | 247493 | 2.57E-05 | Protein_kri1_ |
| RhK5_7634_1361P | Fvb6 | 248330 | 1.94E-05 | Protein_kri1_ |
| RhK5_7634_1361Q | Fvb6 | 248330 | 2.41E-05 | Protein_kri1_ |
| RhMCRND_17190_1696Q | Fvb6 | 262484 | 1.40E-05 | Aspartic_proteinase-like_protein_2,_Precursor |
| RhMCRND_7594_672Q | Fvb6 | 268802 | 5.94E-06 | Protein_U26_ |
| RhK5_12076_566Q | Fvb6 | 317236 | 7.74E-07 | Photosystem_I_reaction_center_subunit_XI,_  chloroplastic_(PSI-L),_Precursor_ |
| Rh12GR_78838_640Q | Fvb6 | 323790 | 4.38E-05 | -- |
| RhK5_1945_356P | Fvb6 | 392934 | 5.32E-05 | DNA-directed_RNA_polymerase_subunit_beta''_  (RNA_polymerase_subunit_beta'')_ |
| RhMCRND_22632_1068P | Fvb6 | 393332 | 4.84E-05 | Exostosin-1_ |
| RhK5_17509_543Q | Fvb6 | 459822 | 1.47E-05 | Eukaryotic_translation_initiation_factor_3_  subunit_B_(eIF3b)_ |
| RhMCRND_293_1349Q | Fvb6 | 612858 | 2.72E-06 | Violaxanthin_de-epoxidase,_chloroplastic  _(AtVxDE),_Precursor_(similar_to) |
| Rh12GR_49737_497Q | Fvb6 | 623780 | 3.28E-05 | -- |
| RhMCRND_10092_296P | Fvb6 | 630966 | 3.55E-06 | Transcription_factor_MYB90_(AtMYB90)_ |
| Rh12GR_32970_131P | Fvb6 | 630966 | 2.31E-05 | Transcription_factor_MYB90_(AtMYB90)_ |
| RhMCRND_10092_296Q | Fvb6 | 630966 | 3.70E-05 | Transcription_factor_MYB90_(AtMYB90)_ |
| RhK5_376_2994P | Fvb6 | 701956 | 5.12E-06 | Probable_UDP-N-acetylglucosamine--peptide_N-acetylglucosaminyltransferase_SEC_ |
| RhK5_376_1591Q | Fvb6 | 701956 | 1.83E-05 | Probable_UDP-N-acetylglucosamine--peptide_N-acetylglucosaminyltransferase_SEC_ |
| RhK5_376_1591P | Fvb6 | 701956 | 4.55E-05 | Probable_UDP-N-acetylglucosamine--peptide_N-acetylglucosaminyltransferase_SEC_ |
| RhK5_160_808Q | Fvb6 | 719356 | 5.49E-05 | Protein_PIR_(AtSRA1)_(similar_to) |
| Rh12GR_34657_1163P | Fvb6 | 738048 | 2.74E-05 | Pentatricopeptide_repeat-containing_protein_ At5g04810,_chloroplastic _(AtPPR4),_Precursor |
| RhK5_8792_536P | Fvb6 | 741285 | 3.14E-05 | Pentatricopeptide_repeat-containing_protein_ At5g04810,_chloroplastic _(AtPPR4),_Precursor |
| RhMCRND_34379_145Q | Fvb6 | 738048 | 4.84E-05 | Myosin_ID_heavy_chain_ |
| RhK5_661_2871P | Fvb6 | 1002381 | 4.85E-05 | Cell_division_cycle_protein_48_homolog_(VCP)_ |
| RhK5_92_2330Q | Fvb6 | 1029476 | 1.38E-05 | Lysine-specific_demethylase_5A_(RBBP-2)_ |
| Rh12GR_5904_1511Q | Fvb6 | 1340822 | 3.15E-05 | Protein_EIN4_(AtEIN4)_ |
| RhK5_7333_2318P | Fvb6 | 16004894 | 3.24E-05 | Gamma-tubulin_complex_component _3_homolog_(x109p) |
| RhK5_285_911Q | Fvb6 | 16300878 | 3.77E-05 | Kinesin-related_protein_11_ |
| RhK5_21702_1402P | Fvb6 | 16502097 | 1.21E-05 | Serine/arginine_repetitive_matrix_protein_2  _(Ser/Arg-related_nuclear_matrix_protein)_ |
| Rh12GR_22762_321P | Fvb6 | 30195732 | 3.42E-05 | E3_ubiquitin-protein_ligase_CIP8_ |
| Rh12GR_22762_321Q | Fvb6 | 30195732 | 5.38E-05 | E3_ubiquitin-protein_ligase_CIP8_ |
| Rh12GR_20064_1031P | Fvb6 | 38202291 | 1.21E-07 | Medium-chain-fatty-acid--CoA_ligase |
| RhMCRND_9549_1730Q | Fvb7 | 2868680 | 1.60E-05 | DNA_endonuclease_ |
