# Supplementary material for: Genome-Wide Association Analysis of the Anthocyanin and Carotenoid Contents of Rose Petals
Source: Front Plant Sci. 2016 Dec 6;7:1798. doi: 10.3389/fpls.2016.01798 (PMC5138216; doi:10.3389/fpls.2016.01798)
Supplement: Table S7 — SNPs significantly associated with carotenoid content and their function, p-value, and effect (untransformed value) on carotenoid content in rose petals. [file Table7.DOCX]

**Table S7.** SNPs significantly associated with carotenoid content and their function, p-value and effect (untransformed value) on carotenoid content in rose petals.

| **Marker** | **Allele** | **Obs** | **Allele** | **Obs** | **Effect** | **p-value** | **function** |
| --- | --- | --- | --- | --- | --- | --- | --- |
| Rh12GR_1204_1323P | A:A | 49 | A:B | 43 | -0.0943 | 1.47E-09 | 30s ribosomal protein s1 |
| Rh12GR_1204_1323Q | A:A | 50 | A:B | 41 | -0.1032 | 1.56E-09 | 30s ribosomal protein s1 |
| Rh12GR_12546_1223Q | B:B | 31 | A:B | 57 | -0.0644 | 4.35E-09 | methyl- -binding domain-containing protein 13-  like isoform x2 |
| Rh12GR_12688_124Q | B:B | 50 | A:B | 43 | -0.1023 | 2.06E-09 | f-box lrr-repeat protein at3g26922-like |
| Rh12GR_13001_501P | A:A | 33 | A:B | 55 | -0.0797 | 1.14E-10 | Acidic_peptide, _Precursor_(probable) |
| Rh12GR_16724_879Q | A:B | 30 | B:B | 56 | -0.0971 | 1.71E-06 | RNA-binding_protein_5-B_(similar_to) |
| Rh12GR_17047_3169Q | A:A | 53 | A:B | 38 | -0.0981 | 1.62E-07 | probable isoleucine--trna cytoplasmic |
| Rh12GR_17126_1100P | A:A | 40 | A:B | 45 | -0.1032 | 4.12E-10 | transcription factor myb86 |
| Rh12GR_1853_1624P | B:B | 61 | A:B | 31 | -0.1526 | 8.02E-10 | sieve element occlusion a |
| Rh12GR_18820_567P | A:A | 48 | A:B | 40 | -0.1629 | 6.40E-11 | ring-h2 finger protein atl21b |
| Rh12GR_1924_1687Q | B:B | 59 | A:B | 32 | -0.0983 | 1.22E-06 | uncharacterized protein LOC101312925 |
| Rh12GR_19272_443P | A:B | 35 | B:B | 56 | -0.1060 | 1.97E-06 | - |
| Rh12GR_19549_1039Q | B:B | 38 | A:B | 51 | 0.1398 | 6.92E-07 | zinc knuckle (cchc-type) family protein |
| Rh12GR_22647_3242P | B:B | 36 | A:B | 49 | -0.0990 | 4.97E-09 | wd repeat-containing protein 7 |
| Rh12GR_23014_1397Q | A:B | 36 | B:B | 54 | -0.0832 | 1.77E-06 | Disease_resistance_protein_At4g27190_probable |
| Rh12GR_23732_124P | B:B | 47 | A:B | 39 | -0.1088 | 3.49E-07 | probable boron transporter 2 |
| Rh12GR_23732_124Q | B:B | 58 | A:B | 26 | -0.1877 | 3.83E-08 | probable boron transporter 2 |
| Rh12GR_25827_1930P | B:B | 42 | A:B | 49 | -0.0719 | 2.25E-09 | e3 ubiquitin-protein ligase ubr7 |
| Rh12GR_2597_1459P | A:A | 49 | A:B | 42 | -0.1237 | 2.77E-10 | protein trichome birefringence-like 23 |
| Rh12GR_2597_1459Q | A:A | 49 | A:B | 41 | -0.1283 | 1.45E-09 | protein trichome birefringence-like 23 |
| Rh12GR_26202_1491P | B:B | 48 | A:B | 40 | -0.0993 | 3.27E-08 | protein far1-related sequence 5-like |
| Rh12GR_26776_1835P | A:B | 35 | B:B | 54 | 0.0986 | 9.34E-07 | cytochrome p450 cyp749a22-like |
| Rh12GR_2834_2977P | B:B | 30 | A:B | 54 | -0.0897 | 1.16E-11 | bifunctional udp-glucose 4-epimerase and  udp-xylose 4-epimerase 1 |
| Rh12GR_28380_1922P | B:B | 50 | A:B | 40 | -0.0959 | 3.56E-07 | sun domain-containing protein 2 isoform x1 |
| Rh12GR_30352_1770Q | B:B | 24 | A:B | 61 | -0.0393 | 3.53E-08 | probable copper-transporting atpase hma5 |
| Rh12GR_31637_1127P | A:A | 46 | A:B | 43 | -0.0971 | 8.39E-09 | gdsl esterase lipase at4g10955 |
| Rh12GR_31637_1127Q | A:A | 50 | A:B | 37 | -0.1007 | 1.31E-06 | GDSL_esterase/lipase_At4g10955_(similar_to) |
| Rh12GR_3624_1035P | B:B | 31 | A:B | 60 | -0.0547 | 1.02E-08 | Adenosine_3'-phospho_5'-phosphosulfate_  transporter_2 |
| Rh12GR_3624_1035Q | B:B | 29 | A:B | 54 | -0.1024 | 2.10E-10 | Adenosine_3'-phospho_5'-phosphosulfate_  transporter_2 |
| Rh12GR_45059_260P | B:B | 50 | A:B | 41 | -0.0955 | 5.01E-09 | phosphatase pp1 isoform x1 |
| Rh12GR_45059_260Q | B:B | 48 | A:B | 40 | -0.1253 | 2.63E-09 | phosphatase pp1 isoform x1 |
| Rh12GR_4587_494P | A:A | 43 | A:B | 48 | -0.0784 | 2.94E-09 | bag family molecular chaperone regulator 1-like |
| Rh12GR_4587_494Q | A:A | 43 | A:B | 50 | -0.0571 | 2.95E-09 | bag family molecular chaperone regulator 1-like |
| Rh12GR_48320_3105P | A:B | 36 | B:B | 56 | -0.1085 | 2.34E-06 | Probable_disease_resistance_RPP8-like_protein_2 |
| Rh12GR_48669_1965P | A:A | 58 | A:B | 27 | -0.1100 | 3.65E-07 | adenine phosphoribosyltransferase 3 |
| Rh12GR_50242_203P | A:B | 35 | B:B | 55 | -0.0619 | 3.82E-08 | F. vesca uncharacterized LOC101293293 |
| Rh12GR_51005_1263P | A:A | 37 | A:B | 53 | -0.0782 | 1.06E-09 | probable beta- -galactosyltransferase 11 |
| Rh12GR_51005_1263Q | A:A | 36 | A:B | 56 | -0.0665 | 2.00E-10 | probable beta- -galactosyltransferase 11 |
| Rh12GR_5175_1076P | B:B | 46 | A:B | 44 | -0.0921 | 2.53E-09 | e3 ubiquitin-protein ligase at3g02290 |
| Rh12GR_5199_576Q | B:B | 48 | A:B | 36 | -0.1034 | 1.19E-06 | heptahelical transmembraneprotein 4-like |
| Rh12GR_54136_228P | B:B | 46 | A:B | 43 | -0.0904 | 6.46E-10 | ---NA--- |
| Rh12GR_54136_228Q | B:B | 49 | A:B | 43 | -0.1057 | 2.24E-10 | ---NA--- |
| Rh12GR_54356_1237P | A:A | 40 | A:B | 49 | -0.0762 | 5.32E-09 | act domain-containing family protein |
| Rh12GR_54356_1237Q | A:A | 41 | A:B | 49 | -0.0854 | 9.89E-10 | act domain-containing family protein |
| Rh12GR_55662_678Q | B:B | 48 | A:B | 42 | -0.0885 | 9.11E-08 | phospho-2-dehydro-3-deoxyheptonate aldolase  chloroplastic-like |
| Rh12GR_56586_394P | B:B | 48 | A:B | 44 | -0.1063 | 5.06E-11 | aquaporin pip1-1 |
| Rh12GR_56586_394Q | B:B | 48 | A:B | 41 | -0.1281 | 2.45E-11 | aquaporin pip1-1 |
| Rh12GR_5673_899P | A:A | 46 | A:B | 46 | -0.0849 | 4.62E-09 | transmembrane protein 64-like |
| Rh12GR_58381_128Q | A:A | 44 | A:B | 46 | -0.0883 | 1.75E-07 | jmjc domain-containing protein 4 |
| Rh12GR_60278_1192P | B:B | 31 | A:B | 57 | -0.0706 | 4.21E-08 | dna-directed rna polymerase ivsubunit 1-like |
| Rh12GR_6046_1211P | A:A | 32 | A:B | 54 | -0.0741 | 1.39E-09 | GDT1-like protein 1, chloroplastic |
| Rh12GR_65691_190Q | A:A | 49 | A:B | 41 | -0.1669 | 2.49E-10 | disease resistance protein (tir-nbs class) |
| Rh12GR_66009_245P | A:B | 31 | B:B | 56 | -0.0681 | 1.82E-07 | ---NA--- |
| Rh12GR_69495_349P | A:A | 48 | A:B | 44 | -0.1329 | 1.47E-06 | - |
| Rh12GR_7292_2456Q | A:A | 52 | A:B | 39 | -0.0517 | 1.31E-06 | Pantothenate_kinase_2_(similar_to) |
| Rh12GR_7364_1628Q | A:A | 54 | A:B | 38 | 0.0872 | 2.16E-06 | Protein_starmaker,_Precursor_(probable) |
| Rh12GR_73809_1022P | B:B | 46 | A:B | 43 | -0.1318 | 3.40E-11 | probable gaba transporter 2 |
| Rh12GR_779_2186Q | B:B | 47 | A:B | 40 | -0.1171 | 3.09E-11 | puromycin-sensitive aminopeptidase isoform x1 |
| Rh12GR_779_512P | A:A | 36 | A:B | 56 | -0.0820 | 6.83E-10 | puromycin-sensitive aminopeptidase isoform x1 |
| Rh12GR_779_512Q | A:A | 36 | A:B | 50 | -0.1012 | 6.67E-10 | puromycin-sensitive aminopeptidase isoform x1 |
| Rh12GR_79799_868P | A:A | 55 | A:B | 37 | -0.1147 | 7.54E-07 | pentatricopeptide repeat-containing protein at5g37570 |
| Rh12GR_82333_268P | A:B | 28 | B:B | 59 | -0.1251 | 1.95E-06 | - |
| Rh12GR_8310_374P | A:B | 32 | B:B | 60 | -0.0909 | 2.19E-06 | hybrid_Trigger_factor_(TF)_(probable) |
| Rh12GR_85205_1084P | A:A | 35 | A:B | 53 | -0.1074 | 4.01E-10 | 1-aminocyclopropane-1-carboxylate synthase |
| Rh12GR_85205_1084Q | A:A | 35 | A:B | 57 | -0.0667 | 3.52E-09 | 1-aminocyclopropane-1-carboxylate synthase |
| Rh12GR_8591_1510Q | B:B | 32 | A:B | 51 | -0.0882 | 2.71E-11 | basic helix-loop-helix dna-binding superfamily  isoform 1 |
| Rh12GR_87307_312Q | A:A | 41 | A:B | 49 | -0.0854 | 6.07E-10 | acyl-coenzyme a:6-aminopenicillanic-acid-  acyltransferase 29 kda subunit |
| Rh12GR_96875_547P | A:A | 32 | A:B | 58 | -0.1071 | 8.89E-12 | probable polygalacturonase |
| Rh12GR_997_501Q | B:B | 56 | A:B | 35 | 0.0591 | 8.12E-08 | dna (cytosine-5)-methyltransferase cmt3 |
| RhK5_1045_1129P | B:B | 48 | A:B | 45 | -0.1101 | 7.51E-12 | endoglucanase 6 |
| RhK5_1045_1129Q | B:B | 47 | A:B | 44 | -0.1285 | 2.43E-12 | endoglucanase 6 |
| RhK5_10810_152Q | B:B | 50 | A:B | 43 | -0.1023 | 2.06E-09 | leucine-rich repeat family isoform 2 |
| RhK5_10852_1179P | A:A | 49 | A:B | 44 | -0.1069 | 1.11E-10 | intracellular protein transport protein USO1-like |
| RhK5_10852_1179Q | A:A | 45 | A:B | 42 | -0.1217 | 2.20E-10 | intracellular protein transport protein USO1-like |
| RhK5_10852_1852P | B:B | 49 | A:B | 44 | -0.0883 | 9.11E-09 | intracellular protein transport protein USO1-like |
| RhK5_10852_1852Q | B:B | 49 | A:B | 41 | -0.1256 | 1.12E-09 | intracellular protein transport protein USO1-like |
| RhK5_10852_924Q | A:B | 54 | B:B | 38 | 0.0665 | 6.29E-07 | intracellular protein transport protein USO1-like |
| RhK5_1098_863P | A:B | 32 | B:B | 60 | -0.0909 | 2.19E-06 | Dentin_sialoprotein,_Precursor_(probable) |
| RhK5_1098_863Q | A:B | 34 | B:B | 57 | -0.0830 | 1.59E-06 | Dentin_sialoprotein,_Precursor_(probable) |
| RhK5_1100_2297P | A:B | 39 | A:A | 52 | 0.0912 | 6.19E-07 | dentin sialophosphoprotein |
| RhK5_1100_2297Q | A:B | 39 | A:A | 51 | 0.0887 | 4.59E-07 | dentin sialophosphoprotein |
| RhK5_11187_125P | B:B | 45 | A:B | 42 | -0.1046 | 9.77E-07 | proline-rich family protein |
| RhK5_11187_125Q | B:B | 45 | A:B | 42 | -0.1101 | 1.14E-07 | proline-rich family protein |
| RhK5_120_1988Q | A:A | 50 | A:B | 42 | -0.1159 | 3.52E-09 | lysine-specific demethylase ref6 |
| RhK5_120_2860P | A:A | 55 | A:B | 32 | -0.1225 | 4.28E-09 | lysine-specific demethylase ref6 |
| RhK5_120_2860Q | A:A | 64 | A:B | 29 | -0.1721 | 3.35E-08 | lysine-specific demethylase ref6 |
| RhK5_120_3163P | B:B | 43 | A:B | 45 | -0.1112 | 2.25E-07 | lysine-specific demethylase ref6 |
| RhK5_120_3163Q | B:B | 45 | A:B | 44 | -0.1070 | 2.37E-07 | lysine-specific demethylase ref6 |
| RhK5_120_3602P | B:B | 64 | A:B | 29 | -0.1721 | 3.35E-08 | lysine-specific demethylase ref6 |
| RhK5_120_3602Q | B:B | 63 | A:B | 28 | -0.1617 | 6.16E-08 | lysine-specific demethylase ref6 |
| RhK5_1365_1221P | B:B | 63 | A:B | 30 | -0.1724 | 4.00E-09 | copper amine oxidase 1-like |
| RhK5_1365_1221Q | B:B | 63 | A:B | 29 | -0.2072 | 1.83E-09 | copper amine oxidase 1-like |
| RhK5_1365_1901Q | A:A | 51 | A:B | 42 | -0.0971 | 9.37E-09 | copper amine oxidase 1-like |
| RhK5_1372_717P | B:B | 45 | A:B | 45 | -0.0993 | 9.65E-11 | protein fam63b-like |
| RhK5_1372_717Q | B:B | 49 | A:B | 40 | -0.1239 | 1.37E-10 | protein fam63b-like |
| RhK5_14592_70P | A:A | 63 | A:B | 29 | -0.1721 | 8.58E-09 | LRR receptor-like serine/threonine-protein kinase  At4g26540 |
| RhK5_14592_70Q | A:A | 61 | A:B | 30 | -0.1520 | 2.20E-08 | LRR receptor-like serine/threonine-protein kinase  At4g26540 |
| RhK5_14907_412P | A:A | 50 | A:B | 43 | -0.1023 | 2.06E-09 | lignin partial |
| RhK5_14907_412Q | A:A | 49 | A:B | 44 | -0.0936 | 5.52E-08 | lignin partial |
| RhK5_1531_1152P | B:B | 52 | A:B | 34 | -0.0897 | 2.53E-07 | uncharacterized protein LOC101312925 |
| RhK5_1531_1152Q | A:B | 35 | B:B | 55 | -0.0759 | 1.88E-06 | Suppressor_protein_SRP40_(probable) |
| RhK5_1609_1178P | B:B | 62 | A:B | 29 | -0.1759 | 4.51E-08 | probable ufm1-specific protease |
| RhK5_1609_1178Q | B:B | 60 | A:B | 29 | -0.1666 | 9.18E-07 | probable ufm1-specific protease |
| RhK5_1614_981P | B:B | 63 | A:B | 28 | -0.1268 | 1.20E-08 | t-complex protein 1 subunit alpha |
| RhK5_1614_981Q | B:B | 65 | A:B | 26 | -0.1067 | 9.22E-07 | t-complex protein 1 subunit alpha |
| RhK5_16428_177P | A:A | 60 | A:B | 31 | -0.1161 | 1.38E-06 | Probable_methyltransferase_TARBP1_(TRP-185)_(similar_to) |
| RhK5_16428_177Q | A:A | 62 | A:B | 22 | -0.1513 | 2.28E-06 | Probable_methyltransferase_TARBP1_(TRP-185)_(similar_to) |
| RhK5_16769_1301Q | A:A | 50 | A:B | 43 | -0.1022 | 2.06E-09 | glutamate--cysteine chloroplastic-like |
| RhK5_16769_510P | B:B | 49 | A:B | 44 | -0.0883 | 9.11E-09 | glutamate--cysteine chloroplastic-like |
| RhK5_16769_510Q | B:B | 50 | A:B | 40 | -0.1661 | 4.08E-10 | glutamate--cysteine chloroplastic-like |
| RhK5_1688_1732P | B:B | 49 | A:B | 43 | -0.1174 | 4.49E-09 | protein trichome birefringence-like 23 |
| RhK5_1688_1732Q | B:B | 47 | A:B | 45 | -0.1010 | 4.10E-10 | protein trichome birefringence-like 23 |
| RhK5_17210_762Q | B:B | 58 | A:B | 33 | -0.1089 | 2.31E-07 | probable phosphoribosylformylglycin-amidine  chloroplastic mitochondrial |
| RhK5_1729_1025Q | A:A | 63 | A:B | 29 | -0.1298 | 2.36E-06 | Pantothenate_kinase_2_(similar_to) |
| RhK5_17744_1032P | A:A | 62 | A:B | 31 | -0.1385 | 1.87E-07 | lrr receptor-like serine threonine-protein kinase hsl2 |
| RhK5_17744_1032Q | A:A | 62 | A:B | 30 | -0.1376 | 3.60E-07 | lrr receptor-like serine threonine-protein kinase hsl2 |
| RhK5_17744_885P | A:A | 59 | A:B | 34 | -0.1124 | 1.83E-07 | lrr receptor-like serine threonine-protein kinase hsl2 |
| RhK5_18146_740P | A:A | 49 | A:B | 40 | -0.1661 | 5.26E-10 | PREDICTED: uncharacterized protein LOC101312440 |
| RhK5_18146_740Q | A:A | 50 | A:B | 41 | -0.1660 | 2.07E-10 | PREDICTED: uncharacterized protein LOC101312440 |
| RhK5_18173_148Q | A:A | 63 | A:B | 27 | -0.1752 | 2.48E-08 | probable pectate lyase 18 |
| RhK5_18173_776Q | B:B | 63 | A:B | 30 | -0.1723 | 4.00E-09 | probable pectate lyase 18 |
| RhK5_18624_1238Q | B:B | 49 | A:B | 39 | -0.0955 | 2.36E-09 | PREDICTED: uncharacterized protein LOC101294263 |
| RhK5_19274_1097P | B:B | 63 | A:B | 24 | -0.2141 | 2.15E-07 | wd repeat-containing protein 74-like |
| RhK5_1999_894P | A:A | 59 | A:B | 32 | -0.1412 | 8.10E-09 | aldehyde dehydrogenase family 2 member  mitochondrial |
| RhK5_1999_894Q | A:A | 59 | A:B | 30 | -0.1667 | 3.05E-09 | aldehyde dehydrogenase family 2 member  mitochondrial |
| RhK5_20022_554P | A:B | 42 | A:A | 46 | -0.1154 | 1.00E-06 | histone-lysine n-methyltransferase clf |
| RhK5_20399_1066Q | A:A | 53 | A:B | 39 | -0.0718 | 1.69E-06 | E3_ubiquitin-protein_ligase_HOS1_(probable) |
| RhK5_20682_1304P | A:A | 58 | A:B | 33 | -0.0818 | 2.39E-06 | Myb-like_protein_J_(probable) |
| RhK5_2071_433P | B:B | 50 | A:B | 43 | -0.1023 | 2.06E-09 | F.vesca signal peptide peptidase-like 2 (LOC101307202) |
| RhK5_2071_433Q | B:B | 50 | A:B | 42 | -0.1249 | 8.49E-10 | F.vesca signal peptide peptidase-like 2 (LOC101307202) |
| RhK5_20798_311P | A:A | 60 | A:B | 31 | -0.1520 | 9.32E-09 | octicosapeptide phox bem1p family protein |
| RhK5_20798_311Q | A:A | 59 | A:B | 27 | -0.1959 | 3.69E-09 | octicosapeptide phox bem1p family protein |
| RhK5_2084_573P | A:B | 38 | B:B | 48 | -0.0719 | 2.18E-06 | Chaperone_protein_dnaJ_13_(AtJ13)_(similar_to) |
| RhK5_2085_1064Q | B:B | 59 | A:B | 28 | -0.1059 | 5.44E-07 | monocopper oxidase-like protein sku5 |
| RhK5_2085_290P | B:B | 56 | A:B | 37 | -0.0731 | 1.02E-06 | monocopper oxidase-like protein sku5 |
| RhK5_2098_875Q | B:B | 65 | A:B | 27 | -0.1615 | 2.37E-07 | peroxisomal membrane protein pex14 |
| RhK5_21321_285Q | A:A | 38 | A:B | 49 | -0.1032 | 5.07E-10 | adenosine monophosphate-protein transferase  and cysteine protease ibpa |
| RhK5_222_1140Q | A:A | 51 | A:B | 42 | -0.0971 | 9.37E-09 | atpase family aaa domain-containing protein 1-a  isoform 1 |
| RhK5_222_1548P | A:B | 41 | B:B | 43 | 0.0812 | 1.96E-06 | ATPase_family_AAA_domain-containing_protein_ 1-A_(probable) |
| RhK5_222_1841P | B:B | 55 | A:B | 28 | -0.1784 | 9.81E-08 | atpase family aaa domain-containing protein 1-a  isoform 1 |
| RhK5_222_1841Q | B:B | 55 | A:B | 35 | -0.1627 | 4.73E-08 | atpase family aaa domain-containing protein 1-a  isoform 1 |
| RhK5_222_2419Q | B:B | 57 | A:B | 36 | -0.1214 | 1.94E-07 | atpase family aaa domain-containing protein 1-a  isoform 1 |
| RhK5_222_2875Q | B:B | 63 | A:B | 29 | -0.1743 | 2.25E-08 | atpase family aaa domain-containing protein 1-a  isoform 1 |
| RhK5_2385_813P | B:B | 60 | A:B | 30 | -0.1764 | 1.34E-08 | atp-dependent zinc metalloprotease |
| RhK5_2385_813Q | B:B | 61 | A:B | 29 | -0.1820 | 2.68E-08 | atp-dependent zinc metalloprotease |
| RhK5_259_2025P | B:B | 50 | A:B | 42 | -0.1046 | 1.03E-09 | myosin heavy chain-related |
| RhK5_259_2025Q | B:B | 50 | A:B | 43 | -0.1023 | 2.06E-09 | myosin heavy chain-related |
| RhK5_259_452P | B:B | 50 | A:B | 42 | -0.1242 | 7.21E-10 | myosin heavy chain-related |
| RhK5_259_452Q | B:B | 50 | A:B | 43 | -0.1023 | 2.06E-09 | myosin heavy chain-related |
| RhK5_2593_1413P | B:B | 61 | A:B | 30 | -0.1330 | 2.40E-08 | serine threonine-protein kinase rio1-like |
| RhK5_264_137Q | B:B | 49 | A:B | 43 | -0.1097 | 5.39E-11 | lysine-specific demethylase 5bisoform x1 |
| RhK5_264_5007P | A:A | 33 | A:B | 54 | -0.1308 | 4.90E-11 | lysine-specific demethylase 5bisoform x1 |
| RhK5_264_5007Q | A:A | 35 | A:B | 58 | -0.0752 | 2.49E-09 | lysine-specific demethylase 5bisoform x1 |
| RhK5_264_5097P | A:A | 32 | A:B | 56 | -0.1013 | 3.71E-11 | lysine-specific demethylase 5bisoform x1 |
| RhK5_264_5097Q | A:A | 34 | A:B | 58 | -0.0778 | 2.42E-09 | lysine-specific demethylase 5bisoform x1 |
| RhK5_264_5394Q | A:A | 45 | A:B | 47 | -0.0688 | 4.88E-09 | lysine-specific demethylase 5bisoform x1 |
| RhK5_2644_952Q | B:B | 53 | A:B | 37 | -0.1172 | 2.28E-07 | quinolinate chloroplastic |
| RhK5_2653_1225Q | A:B | 46 | B:B | 46 | -0.0916 | 1.84E-06 | 1-deoxy-D-xylulose_5-phosphate_reductoisomer-ase,_chloroplastic |
| RhK5_2653_1303P | A:A | 61 | A:B | 24 | -0.1637 | 1.14E-06 | 1-deoxy-d-xylulose 5-phosphate reductoisomerase |
| RhK5_2653_1303Q | A:A | 59 | A:B | 24 | -0.1674 | 2.52E-08 | 1-deoxy-d-xylulose 5-phosphate reductoisomerase |
| RhK5_2847_160P | A:A | 43 | A:B | 43 | -0.1178 | 5.24E-10 | membrane peptidase ydil |
| RhK5_2864_823Q | A:A | 49 | A:B | 43 | -0.0857 | 1.06E-08 | lysophospholipid acyltransferase 1-like |
| RhK5_2968_844P | A:A | 60 | A:B | 32 | -0.1159 | 2.08E-08 | gata transcription factor 26-like |
| RhK5_2968_844Q | A:A | 59 | A:B | 34 | -0.0841 | 1.50E-07 | gata transcription factor 26-like |
| RhK5_2996_1078P | B:B | 50 | A:B | 42 | -0.1243 | 7.21E-10 | tubulin beta chain-like |
| RhK5_2996_1078Q | B:B | 50 | A:B | 41 | -0.1660 | 2.07E-10 | tubulin beta chain-like |
| RhK5_3059_1409P | B:B | 49 | A:B | 39 | -0.1226 | 5.25E-10 | fas-associated factor 2-b |
| RhK5_3059_1409Q | B:B | 50 | A:B | 42 | -0.1014 | 3.02E-09 | fas-associated factor 2-b |
| RhK5_3118_960P | B:B | 64 | A:B | 29 | -0.1721 | 3.35E-08 | probable aspartyl aminopeptidase |
| RhK5_313_2242P | A:A | 43 | A:B | 49 | -0.0778 | 1.48E-07 | probable boron transporter 2 |
| RhK5_313_2337P | A:A | 45 | A:B | 47 | -0.1054 | 1.08E-07 | probable boron transporter 2 |
| RhK5_3176_1256Q | A:B | 33 | B:B | 60 | -0.0854 | 1.71E-06 | - |
| RhK5_3208_462P | A:A | 61 | A:B | 30 | -0.1486 | 5.52E-09 | nuclear control of atpase protein 2-like |
| RhK5_3314_1230Q | A:B | 26 | B:B | 67 | -0.1245 | 1.98E-06 | diponectin_receptor_protein_2_(probable) |
| RhK5_3383_97Q | B:B | 61 | A:B | 28 | -0.1941 | 1.97E-09 | probable lrr receptor-like serine threonine-  protein kinase at4g26540 |
| RhK5_3396_1037Q | B:B | 62 | A:B | 31 | -0.1385 | 1.87E-07 | kinase ht1-like |
| RhK5_3396_623P | B:B | 60 | A:B | 31 | -0.1397 | 1.05E-07 | kinase ht1-like |
| RhK5_3396_623Q | B:B | 59 | A:B | 34 | -0.1141 | 7.76E-09 | kinase ht1-like |
| RhK5_3469_99P | B:B | 63 | A:B | 30 | -0.1723 | 4.00E-09 | sieve element occlusion protein |
| RhK5_3469_99Q | B:B | 64 | A:B | 28 | -0.2163 | 1.31E-08 | sieve element occlusion protein |
| RhK5_3503_549P | A:A | 50 | A:B | 40 | -0.0914 | 6.69E-09 | methyl- -binding domain-containing protein 13  isoform x1 |
| RhK5_3503_549Q | A:A | 46 | A:B | 47 | -0.0818 | 2.87E-09 | methyl- -binding domain-containing protein 13  isoform x1 |
| RhK5_3793_1280P | A:B | 43 | B:B | 47 | -0.1192 | 2.16E-06 | Nicotinate_phosphoribosyltransferase_(NAPRTase)_(similar_to) |
| RhK5_3793_1280Q | A:B | 44 | B:B | 47 | -0.1172 | 1.68E-06 | Nicotinate_phosphoribosyltransferase_(NAPRTase)_(similar_to) |
| RhK5_3895_1162P | B:B | 28 | A:B | 62 | -0.0470 | 2.86E-07 | inactive rhomboid protein 1-like |
| RhK5_3895_1162Q | B:B | 29 | A:B | 63 | -0.0514 | 2.16E-08 | inactive rhomboid protein 1-like |
| RhK5_3982_1257Q | B:B | 46 | A:B | 43 | -0.1279 | 8.02E-11 | protein fizzy-related 2 |
| RhK5_3982_999Q | A:A | 50 | A:B | 40 | -0.1219 | 1.81E-09 | protein fizzy-related 2 |
| RhK5_44_1166P | B:B | 63 | A:B | 30 | -0.1723 | 4.00E-09 | callose synthase 10 |
| RhK5_44_1370P | A:A | 45 | A:B | 47 | -0.1041 | 1.32E-07 | callose synthase 10 |
| RhK5_44_1774Q | B:B | 63 | A:B | 30 | -0.1723 | 4.00E-09 | callose synthase 10 |
| RhK5_44_2489P | A:A | 64 | A:B | 29 | -0.1721 | 3.35E-08 | callose synthase 10 |
| RhK5_44_2489Q | A:A | 64 | A:B | 27 | -0.1662 | 1.22E-07 | callose synthase 10 |
| RhK5_44_2850Q | B:B | 43 | A:B | 46 | -0.1089 | 9.62E-08 | callose synthase 10 |
| RhK5_44_5307P | B:B | 62 | A:B | 29 | -0.1641 | 2.61E-08 | callose synthase 10 |
| RhK5_4506_760P | B:B | 47 | A:B | 46 | -0.0983 | 6.21E-11 | probable rhamnose biosyntheticenzyme 1 |
| RhK5_452_1963P | A:A | 50 | A:B | 42 | -0.1243 | 7.21E-10 | puromycin-sensitive aminopeptidase isoform x1 |
| RhK5_452_438P | A:A | 49 | A:B | 43 | -0.1050 | 2.21E-09 | puromycin-sensitive aminopeptidase isoform x1 |
| RhK5_4806_1112Q | A:A | 52 | A:B | 38 | -0.0698 | 1.17E-06 | serine/arginine repetitive matrix protein 1-like |
| RhK5_4806_58Q | B:B | 58 | A:B | 32 | -0.1022 | 6.27E-07 | serine/arginine repetitive matrix protein 1-like |
| RhK5_4820_815Q | A:A | 45 | A:B | 47 | -0.06982 | 8.73E-08 | ethylene-responsive transcriptionfactor crf2-like |
| RhK5_4932_631P | A:A | 50 | A:B | 42 | -0.1010 | 3.92E-09 | serine racemase |
| RhK5_4932_631Q | A:A | 50 | A:B | 42 | -0.1021 | 3.99E-09 | serine racemase |
| RhK5_4940_1813P | A:A | 54 | A:B | 29 | -0.155 | 1.58E-09 | protein modifier of snc1 1 |
| RhK5_4940_1813Q | A:A | 51 | A:B | 42 | -0.0971 | 9.37E-09 | protein modifier of snc1 1 |
| RhK5_4940_2994Q | A:A | 48 | A:B | 42 | -0.0754 | 1.62E-08 | protein modifier of snc1 1 |
| RhK5_4940_3488P | A:A | 60 | A:B | 31 | -0.1364 | 2.32E-07 | protein modifier of snc1 1 |
| RhK5_4940_3488Q | A:A | 61 | A:B | 28 | -0.1279 | 4.26E-07 | protein modifier of snc1 1 |
| RhK5_4940_3754P | B:B | 64 | A:B | 28 | -0.2163 | 1.31E-08 | protein modifier of snc1 1 |
| RhK5_4940_4304P | A:A | 45 | A:B | 47 | -0.1385 | 5.09E-08 | protein modifier of snc1 1 |
| RhK5_4940_4304Q | A:A | 43 | A:B | 42 | -0.1363 | 2.70E-08 | protein modifier of snc1 1 |
| RhK5_4940_4571Q | A:A | 51 | A:B | 37 | -0.1111 | 6.28E-08 | protein modifier of snc1 1 |
| RhK5_5079_1000Q | A:B | 48 | A:A | 42 | -0.1014 | 6.77E-07 | homeodomain-like superfamily |
| RhK5_5130_176Q | B:B | 62 | A:B | 29 | -0.1714 | 6.12E-09 | probable lrr receptor-like serinethreonine-  protein kinase at4g26540 |
| RhK5_5323_89Q | B:B | 61 | A:B | 31 | -0.1397 | 5.37E-08 | wat1-related protein at3g28050-like |
| RhK5_5377_709P | A:A | 61 | A:B | 23 | -0.1591 | 1.49E-06 | Primary_amine_oxidase,_Precursor_(similar_to) |
| RhK5_5597_889P | B:B | 54 | A:B | 38 | -0.0746 | 3.12E-07 | vesicle-associated protein 1-2-like |
| RhK5_5672_837Q | A:A | 35 | A:B | 52 | -0.1000 | 2.01E-09 | short-chain dehydrogenase tic chloroplastic-like |
| RhK5_5672_897P | A:A | 49 | A:B | 42 | -0.1084 | 3.68E-09 | short-chain dehydrogenase tic chloroplastic-like |
| RhK5_5672_897Q | A:A | 50 | A:B | 43 | -0.1023 | 2.06E-09 | short-chain dehydrogenase tic chloroplastic-like |
| RhK5_5712_264P | B:B | 47 | A:B | 45 | -0.1064 | 2.25E-11 | reticulon-like protein b2 |
| RhK5_5712_264Q | B:B | 50 | A:B | 40 | -0.0988 | 6.72E-09 | reticulon-like protein b2 |
| RhK5_5712_567P | A:A | 48 | A:B | 43 | -0.1068 | 1.72E-10 | reticulon-like protein b2 |
| RhK5_5839_826Q | A:A | 67 | A:B | 25 | -0.1751 | 5.74E-07 | glutamate dehydrogenase 2 |
| RhK5_5862_192P | A:A | 59 | A:B | 25 | -0.1570 | 9.06E-08 | vegetative cell wall protein gp1-like isoform x2 |
| RhK5_6041_514P | A:B | 44 | B:B | 46 | -0.1002 | 1.19E-06 | hypothetical_protein |
| RhK5_6072_829P | A:A | 49 | A:B | 41 | -0.1632 | 2.78E-11 | serine threonine-proteinphosphatase pp1 |
| RhK5_6072_829Q | A:A | 50 | A:B | 43 | -0.1023 | 2.06E-09 | serine threonine-proteinphosphatase pp1 |
| RhK5_6118_534P | A:A | 49 | A:B | 44 | -0.1069 | 1.11E-10 | 2-c-methyl-d-erythritol -cyclodiphosphate  chloroplastic |
| RhK5_6118_534Q | A:A | 49 | A:B | 38 | -0.1594 | 1.19E-10 | 2-c-methyl-d-erythritol -cyclodiphosphate  chloroplastic |
| RhK5_6336_579P | A:A | 61 | A:B | 27 | -0.1650 | 1.31E-07 | ubiquitin-conjugating enzymee2-23 kda-like |
| RhK5_634_2783P | A:A | 45 | A:B | 46 | -0.0871 | 4.26E-10 | kinesin-4 isoform x1 |
| RhK5_634_2783Q | A:A | 49 | A:B | 44 | -0.1069 | 1.11E-10 | kinesin-4 isoform x1 |
| RhK5_6518_685P | B:B | 49 | A:B | 44 | -0.1069 | 1.11E-10 | nucleoside diphosphate kinase chloroplastic  mitochondrial-like |
| RhK5_6518_909P | B:B | 39 | A:B | 47 | -0.0854 | 5.49E-10 | nucleoside diphosphate kinase chloroplastic  mitochondrial-like |
| RhK5_6590_242P | A:A | 48 | A:B | 40 | -0.1183 | 7.96E-10 | tetraspanin-10 isoform x1 |
| RhK5_6590_242Q | A:A | 49 | A:B | 42 | -0.1262 | 8.00E-10 | tetraspanin-10 isoform x1 |
| RhK5_674_689Q | A:B | 44 | B:B | 47 | -0.1189 | 1.55E-06 | Filament-like_plant_protein_7_(AtFPP7) _(probable) |
| RhK5_6745_668P | B:B | 50 | A:B | 43 | -0.1023 | 2.06E-09 | transmembrane protein 64-like |
| RhK5_6745_668Q | B:B | 50 | A:B | 42 | -0.1243 | 7.21E-10 | transmembrane protein 64-like |
| RhK5_6879_950Q | A:A | 47 | A:B | 41 | -0.0921 | 1.38E-08 | dna-binding protein escarola |
| RhK5_710_2921P | B:B | 45 | A:B | 45 | -0.1018 | 2.99E-07 | nudix hydrolase 3 |
| RhK5_7140_634P | A:A | 64 | A:B | 29 | -0.1721 | 3.35E-08 | M. notabilis wd40 repeat-containing protein 85 |
| RhK5_718_1024P | A:A | 49 | A:B | 43 | -0.1048 | 1.27E-09 | probable lrr receptor-like serine threonine-protein kinase at1g12460 |
| RhK5_718_1024Q | A:A | 50 | A:B | 41 | -0.1285 | 4.29E-10 | probable lrr receptor-like serine threonine-protein kinase at1g12460 |
| RhK5_718_383Q | A:A | 48 | A:B | 43 | -0.0983 | 5.12E-10 | probable lrr receptor-like serine threonine-proteinkinase at1g12460 |
| RhK5_718_563P | B:B | 45 | A:B | 47 | -0.1194 | 4.24E-11 | probable lrr receptor-like serine threonine-protein kinase at1g12460 |
| RhK5_718_563Q | B:B | 45 | A:B | 47 | -0.0999 | 1.65E-10 | probable lrr receptor-like serine threonine-protein kinase at1g12460 |
| RhK5_7183_723Q | A:A | 50 | A:B | 42 | -0.1010 | 3.92E-09 | glutathione s-transferase t1-like |
| RhK5_7274_334P | A:A | 38 | A:B | 53 | -0.0724 | 3.10E-08 | nudix hydrolase chloroplastic |
| RhK5_7274_334Q | A:A | 36 | A:B | 54 | -0.0754 | 2.28E-08 | nudix hydrolase chloroplastic |
| RhK5_7361_296P | B:B | 50 | A:B | 40 | -0.1681 | 4.00E-10 | abc transporter c familymember 10-like |
| RhK5_7361_758P | B:B | 49 | A:B | 44 | -0.1069 | 1.11E-10 | abc transporter c familymember 10-like |
| RhK5_7361_758Q | B:B | 42 | A:B | 45 | -0.0933 | 5.36E-10 | abc transporter c familymember 10-like |
| RhK5_7401_423P | B:B | 63 | A:B | 27 | -0.1440 | 1.80E-07 | iron-sulfur cluster assembly protein 1-like |
| RhK5_7404_235P | A:A | 49 | A:B | 37 | -0.1710 | 1.82E-10 | probable phospholipid hydroperoxide glutathione  peroxidase |
| RhK5_7461_450P | B:B | 61 | A:B | 28 | -0.1631 | 2.07E-08 | nucleoside diphosphate kinase chloroplastic |
| RhK5_7528_1015P | B:B | 61 | A:B | 23 | -0.1779 | 2.68E-09 | nucleic acid- ob-fold-like proteinisoform 1 |
| RhK5_7528_1239Q | A:A | 61 | A:B | 30 | -0.1488 | 2.57E-07 | nucleic acid- ob-fold-like proteinisoform 1 |
| RhK5_7624_963P | B:B | 34 | A:B | 55 | -0.1360 | 1.39E-09 | e3 sumo-protein ligase pli1 |
| RhK5_7755_2216P | B:B | 42 | A:B | 42 | -0.121 | 5.10E-08 | PREDICTED: uncharacterizedprotein LOC101297256 |
| RhK5_7755_2216Q | B:B | 44 | A:B | 47 | -0.0977 | 5.77E-09 | PREDICTED: uncharacterizedprotein LOC101297256 |
| RhK5_7755_2322P | B:B | 60 | A:B | 27 | -0.1877 | 7.02E-09 | PREDICTED: uncharacterizedprotein LOC101297256 |
| RhK5_7755_559P | B:B | 49 | A:B | 36 | -0.0850 | 6.34E-07 | PREDICTED: uncharacterizedprotein LOC101297256 |
| RhK5_7755_559Q | B:B | 50 | A:B | 39 | -0.0962 | 1.62E-08 | PREDICTED: uncharacterizedprotein LOC101297256 |
| RhK5_78_3929P | A:B | 32 | B:B | 59 | -0.0849 | 1.36E-06 | Dentin_sialoprotein,_Precursor_(probable) |
| RhK5_7933_721Q | A:A | 49 | A:B | 40 | -0.1283 | 7.26E-10 | high mobility group b protein 6-like |
| RhK5_8300_365P | A:A | 61 | A:B | 32 | -0.1346 | 4.96E-08 | probable kinetochore protein spc25 |
| RhK5_8300_667P | A:A | 63 | A:B | 30 | -0.1723 | 4.00E-09 | probable kinetochore protein spc25 |
| RhK5_8300_667Q | A:A | 63 | A:B | 30 | -0.1493 | 1.43E-07 | probable kinetochore protein spc25 |
| RhK5_8330_720Q | A:A | 64 | A:B | 23 | -0.1580 | 1.52E-06 | Brain_protein_44-like_protein_(similar_to) |
| RhK5_8375_403P | B:B | 59 | A:B | 34 | -0.1102 | 4.33E-08 | f-box protein skip8-like |
| RhK5_8375_403Q | B:B | 59 | A:B | 32 | -0.1361 | 4.20E-09 | f-box protein skip8-like |
| RhK5_8430_356P | B:B | 47 | A:B | 36 | -0.1172 | 7.91E-11 | mitogen-activated proteinkinase yoda |
| RhK5_8430_356Q | B:B | 49 | A:B | 40 | -0.1601 | 3.22E-10 | mitogen-activated proteinkinase yoda |
| RhK5_879_361P | B:B | 49 | A:B | 43 | -0.1024 | 8.46E-10 | disease resistance protein (tir-nbs class) |
| RhK5_879_361Q | B:B | 48 | A:B | 43 | -0.0960 | 3.06E-10 | disease resistance protein (tir-nbs class) |
| RhK5_8798_1344P | A:A | 48 | A:B | 43 | -0.1054 | 9.64E-10 | probable gaba transporter 2 |
| RhK5_8852_593P | B:B | 58 | A:B | 33 | -0.0968 | 9.42E-07 | pentatricopeptide repeat-containing protein  chloroplastic |
| RhK5_8852_671P | A:B | 31 | B:B | 61 | -0.0932 | 2.29E-06 | Pentatricopeptide_repeat-containing_protein_ At4g18975,_chloroplastic,_Precursor_(probable) |
| RhK5_9004_989P | A:A | 60 | A:B | 28 | -0.1338 | 3.51E-08 | PREDICTED: uncharacterized protein LOC101309833 |
| RhK5_9004_989Q | A:A | 63 | A:B | 29 | -0.1721 | 8.58E-09 | PREDICTED: uncharacterized protein LOC101309833 |
| RhK5_9065_606P | B:B | 50 | A:B | 42 | -0.1014 | 3.02E-09 | dna repair helicase xpb1 |
| RhK5_9077_390Q | B:B | 61 | A:B | 31 | -0.1445 | 2.90E-08 | e3 ubiquitin-protein ligase march10 |
| RhK5_9169_598P | A:A | 50 | A:B | 39 | -0.1041 | 1.26E-09 | palmitoyl-monogalactosyldiacyl-glycerol delta-7  chloroplastic-like |
| RhK5_9169_598Q | A:A | 48 | A:B | 41 | -0.1021 | 4.05E-09 | palmitoyl-monogalactosyldiacyl-glycerol delta-7  chloroplastic-like |
| RhK5_9287_519Q | A:A | 61 | A:B | 23 | -0.2588 | 1.39E-09 | ap-1 complex subunit mu-2-like |
| RhK5_9287_657P | A:A | 64 | A:B | 28 | -0.1691 | 6.10E-08 | ap-1 complex subunit mu-2-like |
| RhK5_9334_591Q | A:A | 64 | A:B | 27 | -0.1999 | 1.49E-07 | PREDICTED: uncharacterized protein  LOC105351200 isoform X1 |
| RhK5_950_2022Q | B:B | 49 | A:B | 44 | -0.0956 | 7.85E-10 | probable inactive receptor kinase at4g23740 |
| RhK5_958_2150Q | B:B | 61 | A:B | 29 | -0.1792 | 2.51E-09 | programmed cell death protein 4-like |
| RhK5_958_518Q | B:B | 60 | A:B | 31 | -0.1413 | 5.78E-08 | programmed cell death protein 4-like |
| RhK5_958_950P | A:A | 63 | A:B | 26 | -0.1681 | 2.94E-07 | programmed cell death protein 4-like |
| RhK5_958_950Q | A:A | 57 | A:B | 31 | -0.1508 | 1.05E-08 | programmed cell death protein 4-like |
| RhK5_9739_1172P | B:B | 41 | A:B | 51 | -0.0705 | 8.27E-07 | armadillo repeat-containing kinesin-like protein 3 |
| RhK5_9801_542Q | A:A | 50 | A:B | 42 | -0.1047 | 1.65E-09 | histone deacetylase complexsubunit sap18 |
| RhMCRND_10438_726Q | B:B | 34 | A:B | 50 | -0.0250 | 3.66E-10 | Mitogen-activated_protein_kinase_ANP1_(similar) |
| RhMCRND_10913_176Q | A:A | 50 | A:B | 36 | 0.0380 | 1.58E-07 | f-box protein skip16-like |
| RhMCRND_12376_549P | A:B | 33 | A:A | 54 | 0.0320 | 9.56E-07 | protein lsm12 homolog a |
| RhMCRND_12648_158P | B:B | 56 | A:B | 36 | 0.0276 | 7.06E-07 | heme-binding protein 2 |
| RhMCRND_12933_253P | A:A | 46 | A:B | 47 | -0.1171 | 1.94E-06 | Nicotinate_phosphoribosyltransferase_(NAPRTase)_(similar_to) |
| RhMCRND_14427_132Q | B:B | 43 | A:B | 43 | -0.0313 | 3.87E-07 | signal recognition particle receptor subunit beta-like |
| RhMCRND_14720_280P | B:B | 73 | A:B | 20 | -0.0326 | 1.24E-07 | probable glycosyltransferase at3g07620 |
| RhMCRND_16521_257Q | B:B | 39 | A:B | 51 | 0.0452 | 2.80E-11 | Aquaporin_PIP1-3_(AtPIP1)_(putative) |
| RhMCRND_16630_397Q | B:B | 41 | A:B | 43 | 0.0875 | 1.75E-07 | protein misato homolog 1 isoform x1 |
| RhMCRND_2281_1034P | A:A | 33 | A:B | 54 | -0.0187 | 2.29E-06 | Protein_EXECUTER_1,_chloroplastic,_Precursor_(probable) |
| RhMCRND_2772_1314P | A:A | 35 | A:B | 53 | -0.0240 | 3.71E-09 | PREDICTED: uncharacterized protein LOC101304671 |
| RhMCRND_2772_1314Q | A:A | 35 | A:B | 56 | 0.0384 | 1.61E-09 | PREDICTED: uncharacterized protein LOC101304671 |
| RhMCRND_30574_169Q | A:A | 33 | A:B | 53 | -0.0684 | 2.44E-10 | probable GABA transporter 2 |
| RhMCRND_3202_1164P | A:A | 33 | A:B | 59 | -0.0221 | 1.95E-07 | hydroxyproline-rich glycoproteinfamily protein |
| RhMCRND_3606_439P | A:A | 34 | A:B | 56 | -0.0543 | 2.60E-10 | intracellular protein transport protein USO1-like |
| RhMCRND_3606_439Q | A:B | 54 | A:A | 31 | -0.0491 | 6.85E-09 | intracellular protein transport protein USO1-like |
| RhMCRND_3606_99Q | B:B | 36 | A:B | 49 | -0.0991 | 6.15E-11 | intracellular protein transport protein USO1-like |
| RhMCRND_4363_1438P | A:B | 29 | A:A | 61 | -0.0108 | 2.22E-08 | ring-h2 finger protein atl46 |
| RhMCRND_4363_1438Q | A:B | 31 | A:A | 59 | -0.0159 | 3.52E-09 | ring-h2 finger protein atl46 |
| RhMCRND_4773_326Q | A:B | 29 | B:B | 58 | 0.0814 | 2.60E-06 | Galactose_oxidase_(GAO),_Precursor_(probable) |
| RhMCRND_4851_1084Q | B:B | 48 | A:B | 44 | -0.0033 | 1.16E-10 | gene09984-v1.0-hybrid_Cytochrome_ P450  _71A24_(similar_to) |
| RhMCRND_54_4226P | A:A | 38 | A:B | 53 | 0.0675 | 1.95E-06 | Protein_TIME_FOR_COFFEE_(probable) |
| RhMCRND_5501_1048P | A:A | 44 | A:B | 47 | -0.0244 | 6.66E-08 | vignain |
| RhMCRND_61_1846P | A:A | 43 | A:B | 43 | 0.0121 | 2.81E-07 | callose synthase 10 |
| RhMCRND_61_1846Q | A:A | 44 | A:B | 43 | -0.0127 | 1.24E-07 | callose synthase 10 |
| RhMCRND_6174_780P | A:B | 28 | B:B | 64 | -0.1132 | 1.59E-06 | Protein_MAM3_(probable) |
| RhMCRND_633_2817P | A:B | 44 | B:B | 47 | -0.1181 | 1.53E-06 | Filament-like_plant_protein_7_(AtFPP7) _(probable) |
| RhMCRND_633_2817Q | A:B | 44 | B:B | 47 | -0.1183 | 2.55E-06 | Filament-like_plant_protein_7_(AtFPP7) _(probable) |
| RhMCRND_6429_545P | B:B | 43 | A:B | 42 | 0.0230 | 1.67E-08 | probable cyclic nucleotide-gatedion channel 16 |
| RhMCRND_645_325P | A:B | 28 | B:B | 60 | -0.0793 | 2.21E-09 | probable -trehalose-phosphate synthase |
| RhMCRND_6639_1184P | A:A | 58 | A:B | 34 | 0.0191 | 2.01E-08 | gdp-l-fucose synthase 2 |
| RhMCRND_6639_1184Q | A:A | 58 | A:B | 29 | 0.0047 | 8.57E-09 | gdp-l-fucose synthase 2 |
| RhMCRND_6711_944P | A:B | 26 | B:B | 61 | -0.0456 | 2.57E-08 | PREDICTED: uncharacterized protein LOC101310411 |
| RhMCRND_7759_1092P | A:A | 41 | A:B | 51 | 0.0243 | 1.07E-09 | intracellular protein transport protein USO1-like |
| RhMCRND_7759_1092Q | A:A | 38 | A:B | 52 | -0.0146 | 7.97E-10 | intracellular protein transport protein USO1-like |
| RhMCRND_8776_110Q | A:B | 39 | B:B | 52 | 0.0234 | 3.76E-07 | rs-containing zinc finger protein 21 isoform 1 |
| RhMCRND_9229_1627P | A:B | 35 | B:B | 50 | -0.0002 | 2.52E-07 | rna-binding family isoform 1 |
| RhMCRND_9229_1627Q | B:B | 53 | A:B | 32 | -0.0318 | 7.87E-07 | rna-binding family isoform 1 |
| RhMCRND_933_395P | A:B | 32 | B:B | 61 | 0.0108 | 5.08E-09 | upf0392 protein rcom_0530710;  Glycosyltransferase family 92 |
| RhMCRND_933_395Q | A:B | 31 | B:B | 59 | -0.0012 | 1.36E-08 | upf0392 protein rcom_0530710;  Glycosyltransferase family 92 |
| RhMCRND_933_874P | A:B | 30 | B:B | 61 | 0.0599 | 1.16E-09 | upf0392 protein rcom_0530710;  Glycosyltransferase family 92 |
| RhMCRND_9528_922P | A:B | 33 | B:B | 54 | -0.0432 | 2.39E-06 | Transcription_factor_bHLH150_(bHLH_150)_(probable) |
| RhMCRND_9528_922Q | B:B | 55 | A:B | 33 | 0.0594 | 2.69E-07 | transcription factor par2 |
| RhMCRND_9667_858P | A:B | 35 | B:B | 55 | -0.0163 | 7.63E-08 | nucleobase-ascorbate transporter 6-like |
| RhMCRND_9667_858Q | A:B | 35 | B:B | 54 | -0.0529 | 2.94E-07 | nucleobase-ascorbate transporter 6-like |
