# Supplementary material for: Genome-Wide Association Analysis of the Anthocyanin and Carotenoid Contents of Rose Petals
Source: Front Plant Sci. 2016 Dec 6;7:1798. doi: 10.3389/fpls.2016.01798 (PMC5138216; doi:10.3389/fpls.2016.01798)
Supplement: Table S8 — Abbreviations for known transcription factors, carotenoid and anthocyanin biosynthetic pathway genes. [file Table8.DOCX]

Table S 8. Abbreviations for known transcription factors, carotenoid and anthocyanin biosynthetic pathway genes.

| Carotenoids |  |
| --- | --- |
| Pmzeaep | *Prunus mume* - zeaxanthin epoxidase |
| RH_PDS | *Rosa hybrid cultivar* - phytoene desaturase |
| Paphydes | *Prunus armeniaca* - phytoene desaturase |
| Md-CCD4 | *Malus x domestica* - probable carotenoid cleavage dioxygenase 4 |
| Rd_CCD4 | *Rosa x damascena* - carotenoid cleavage dioxygenase |
| Ej_CRTISO | *Eribotrya japonica* - carotenoid isomerase |
| CCD4like | *Malus x domestica* - probable carotenoid cleavage dioxygenase 4 |
| Pp_CCD4a | *Prunus persica* - cleavage dioxygensae 4a |
| Anthocyanins |  |
| Pa_CHS2 | *Prunus avium* - chalcone synthase 2 |
| Pp_Hy5 | *Prunus persica* - transcription factor HY5 |
| Rh_DFR | *Rosa hybrid cultivar* - dyhydroflavonol 4-reductase |
| RhGT1-3 | *Rosa hybrid cultiva*r - anthocyanidin 5,3-O-glucosyltransferase |
| Mh_MYB16 | *Malus hybrid cultivar* - transcription factor Myb16 |
| Pyr_bHLH | *Pyrus pyrifolia* - basic helix-loop-helix transcription factor (bHLH) |
| RhA3GT | *Rosa hybrid cultivar* - anthocyanin 3-glucosyltransferase |
| Rh_R2R3Mybs4 | *Rosa hybrid cultivar* - transcription factor R2R3Mybs4 |
| Pa_Myb10 | *Prunus avium* - transcription factor Myb10 |
| Mh_MYB3 | *Malus hybrid cultivar* - transcription factor MYB3 |
| Pa_F3'H | *Prunus avium* - flavonoid-3'-hydroxylase |
| Md_ANR2a | *Malus x domestica* - anthocyanidin reductase 2 (ANR2a) |
| Md_GST-L | *Malus x domestica* - glutathione *S*-transferase (GST-like protein) |
| Md_AR1 | *Malus x domestica* - anthocyanidin reductase 1 |
| Md_F3'H | *Malus x domestica* - flavonoid-3'-hydroxylase |
| Md_Myb1 | *Malus x domestica* - transcription factor Myb1 |
| Pyr_PAL | *Pyrus pyrifolia* - phenylalanine ammonia lyase |
| Rh_R2R3Myb6 | *Rosa hybrid cultivar* - transcription factor R2R3Myb6 |
| Pp_bHLH3 | *Prunus persica* - basic helix-loop-helix transcription factor (bHLH) |
